# Autologous micrograft accelerates endogenous wound healing response through ERK-induced cell migration

**DOI:** 10.1038/s41418-019-0433-3

**Published:** 2019-10-25

**Authors:** Martina Balli, Francesca Vitali, Adrian Janiszewski, Ellen Caluwé, Alvaro Cortés-Calabuig, Sebastien Carpentier, Robin Duelen, Flavio Ronzoni, Lukas Marcelis, Francesca Maria Bosisio, Riccardo Bellazzi, Aernout Luttun, Maria G. Cusella De Angelis, Gabriele Ceccarelli, Frederic Lluis, Maurilio Sampaolesi

**Affiliations:** 1 Department of Development and Regeneration, Stem Cell Institute, KU Leuven, B-3000 Leuven, Belgium; 2 Human Anatomy Unit, Department of Public Health, Experimental and Forensic Medicine, University of Pavia, Pavia, Italy; 3 Center for Biomedical Informatics and Biostatistics, The University of Arizona Health Sciences, Tucson, AZ USA; 4 Department of Medicine, College of Medicine, The University of Arizona, Tucson, AZ USA; 5 Department of Cardiovascular Sciences, Centre for Molecular and Vascular Biology, KU Leuven, B-3000 Leuven, Belgium; 6 Genomics Core Leuven, Centre for Human Genetics KU Leuven, 3000 Leuven, Belgium; 7 Facility for SYstems BIOlogy based MAss sepctrometry, KU Leuven, 3000 Leuven, Belgium; 8 Center for Health Technologies (CHT), University of Pavia, Pavia, Italy; 9 Translational Cell and Tissue Research Lab, Department of Imaging and Pathology, KU Leuven, Leuven, Belgium; 10 Department of Electrical, Computer and Biomedical Engineering, and Centre for Health Technologies (CHT), University of Pavia, Pavia, Italy

**Keywords:** Molecular biology, Proteomics

## Abstract

Defective cell migration causes delayed wound healing (WH) and chronic skin lesions. Autologous micrograft (AMG) therapies have recently emerged as a new effective and affordable treatment able to improve wound healing capacity. However, the precise molecular mechanism through which AMG exhibits its beneficial effects remains unrevealed. Herein we show that AMG improves skin re-epithelialization by accelerating the migration of fibroblasts and keratinocytes. More specifically, AMG-treated wounds showed improvement of indispensable events associated with successful wound healing such as granulation tissue formation, organized collagen content, and newly formed blood vessels. We demonstrate that AMG is enriched with a pool of WH-associated growth factors that may provide the starting signal for a faster endogenous wound healing response. This work links the increased cell migration rate to the activation of the extracellular signal-regulated kinase (ERK) signaling pathway, which is followed by an increase in matrix metalloproteinase expression and their extracellular enzymatic activity. Overall we reveal the AMG-mediated wound healing transcriptional signature and shed light on the AMG molecular mechanism supporting its potential to trigger a highly improved wound healing process. In this way, we present a framework for future improvements in AMG therapy for skin tissue regeneration applications.

## Introduction

Traumatic skin injuries and impaired wound healing (WH) lead to a widespread public problem, hindering patient quality of life and contributing to mortality.

The autologous micrograft (AMG) technique is an emerging therapy for skin injuries that is able to improve WH, minimizing scar formation, and represents an affordable alternative to traditional skin grafting [1–4]. Using a sterile tissue destroyer, healthy autologous tissues are grinded into freshly available micro-tissue grafts [5]. AMG can be applied directly to the area of injury and it has been reported to ameliorate tissue regeneration in dehiscent wounds [3], in traumatic wounds associated with hypertrophic skin and also in cardiac scars [1, 2]. Most importantly, the AMG treatment that consists of a micrograft drawn directly from the patient, allows the wound coverage with a minimal amount of donor tissue and a limited risk of tissue rejection. However, neither the AMG components nor the putative molecular mechanisms activated upon treatment have yet been studied.

WH is a well-orchestrated process that involves the sequential collaborative efforts of multiple cell types, extracellular matrix (ECM) components, growth factors, and signaling pathways. In the first stage of WH, cytokines and growth factors initiate the wound repair process regulating the inflammatory response. Several studies have listed epidermal growth factor (EGF), basic fibroblast growth factor (bFGF), insulin-like growth factor (IGF), platelet-derived growth factor (PDGF) and transforming growth factor beta (TGF-β) as fundamental growth factors that trigger WH response [6]. Upon the activation of intracellular signaling pathways, like Mitogen-Activated Protein Kinase (MAPK) signaling, WH proceeds further. The activation of MAPK signaling drives cell proliferation/migration of fibroblasts and keratinocytes and differentiation of fibroblast into myofibroblasts, which are crucial events involved in the WH process [7, 8]. Cell contact and soluble molecules are essential for the cross talk between these cell types [9]. Mainly, fibroblasts are responsible for the secretion of extracellular enzymes [10], which allows ECM remodeling, cell migration, re-epithelialization and finally, wound closure.

In this work, we shed light on the molecular mechanisms of AMG-based treatment by providing evidence that this therapy enhances in vivo WH by promoting re-epithelialization and angiogenesis. We show that the AMG tissue extract is enriched with pro-motility growth factors implicated in the initiation of WH, including bFGF and IGF-I. Furthermore, by comparative transcriptome analysis followed by Protein–Protein Interaction (PPI) network analysis of AMG-treated primary fibroblasts, we identify cell migration and ERK/MAPK signaling pathway as essential events in the AMG-mediated WH response. We demonstrate that AMG treatment enhances ERK-dependent MMP gene expression and extracellular enzymatic activity in both keratinocytes and primary fibroblasts leading to accelerated in vitro scratch closure and in vivo WH. Accordingly, inhibition of either ERK phosphorylation or MMP enzymatic activity significantly revert the AMG-mediated WH effects, revealing the molecular mechanisms behind AMG and thereby providing the groundwork for future advances in micrograft therapies for improved WH.

## Materials and methods

### Cell culture

Mouse primary fibroblasts were isolated from tail tips (0.5 cm) of C57BL/6 mouse (4–6 weeks old) model under anesthesia (isoflurane). Primary fibroblasts and keratinocytes were cultured in DMEM GlutaMax (Ca No. 61965-026, Gibco Life Science, Grand island) and Dulbecco’s modified Eagles’ medium (DMEM) respectively. Cell media were supplemented with 10% of fetal bovine serum, 1% of penicillin-streptomycin, 1% of L-glutamine, 1% of sodium pyruvate and 1% of nonessential amino acids. The human keratinocyte (HaCaT) cell line was grown. Inhibitory drugs for MEK (PD0325901 1 μM) and MMP activity (Actinonin 20 μM) were applied to both scratched monolayers and 60–70% primary fibroblasts and keratinocytes (HaCaT).

### Preparation of autologous micrografts

AMGs were obtained from skin murine biopsies (1 cm^2^, collected from the back of the animals) using a sterile tissue destroyer (Rigeneracons*®*) [5]. Tissues were re-suspended in 1 mL PBS, disrupted, and filtered through 70 μm filter membranes (at a final protein concentration of 5 μg/μL). The extracted AMG were then diluted 1:10 in growth media and applied to in vitro scratch wound assay, 60–70% confluent dermal and epithelial cells as well as to in vivo wounds.

### Cell scratch assay

Mouse primary fibroblasts were plated at a density of 3500 cell/cm^2^. Cells at passages 4–6 were used in this study. HaCaT cells were plated at a density of 10,000 cell/cm^2^. After reaching confluence, a scratch was performed on the cell monolayer using a pipette tip, resembling an artificial in vitro wound [11].

### XTT cell survival assay

The in vitro cell survival was assessed, evaluating metabolic activity and micrograft-related sensibility in primary murine fibroblasts exposed to the AMG, following manufacturer instructions (Thermo Fisher Scientific). Cells were plated at a density of 10^5^cell/well, in 96 multi-well culture plates, in 100 µL of Growth medium in which AMGs were diluted (1:10), followed by different time periods of treatment.

### Cell cycle and viability assays

Changes in the cell cycle were assessed using the EdU - 5-ethynyl-2’-deoxyuridine assay (Thermo Fisher Scientific). 60–70% confluent cells were treated with 5 and 12 h AMG treatments or with vehicle (PBS). After each time point, cells were incubated at 37 °C with EdU 10 μM for 2 h [12]. The samples were then acquired by Flow Cytometry (BD-Biosciences) and analyzed by FlowJo software. Ki67 staining was used to evaluate changes in cell proliferation of 60–70% confluent MG-treated human keratinocytes. After 5 h MG-treatment, cells were harvested, fixed with ice-cold 70% ETOH. Blocking step was performed using 5% donkey serum for 30 min at RT. Cells were then pelleted and incubated with purified mouse anti-Ki67 primary antibody (1:100 - Ca No. 556003, BD Pharmingen^TM^) for 30 min at RT. After washes, cells were incubated with AlexaFluor 488 donkey anti-mouse (1:450 in PBS 5% FBS – Thermo Fisher) for 20 min in the dark. Afterward, cells were resuspended in 500 μl of PBS in which PI and RNaseA were added for 30 min at RT. The samples were then acquired by Flow Cytometry (BD-Biosciences) and analyzed by FlowJo software. Cell viability was evaluated by flow cytometry (BD-Biosciences) using the fixable viability stain 660.

### In vitro and in vivo immunohistochemistry and immunofluorescence staining

Scratched fibroblasts were fixed 20’with 4% PFA at RT, permeabilized with 1% BSA, 0,2% Triton-X in PBS, washed and blocked with donkey serum 40 min at RT. On the other hand, 60–70% confluent cells were used to evaluate AMG-dependent cell proliferation effects. As well, murine biopsies from the wound area were collected and further processed for paraffin embedding. The primary antibodies used in this study were Mouse monoclonal anti-actin α-Smooth Muscle - Cy3™ (1:200, C6198, Sigma Aldrich) and Ki67 monoclonal antibody (SP6) (Cat No. MA5-14520 – 1:100). Cells were incubated with secondary-AlexaFluor antibody for 1 h at RT and washed. Hoechst 33342 (1:10000) was used for the nuclear counterstaining. Samples were mounted using FluorSave™ mounting medium (EDM Millipore). Wound biopsies were paraffin-embedded. CD31 (1:500, BD557355, Pharmingen), actin α-Smooth Muscle - Cy3™ (1:200, C6198, Sigma Aldrich), p-ERK (1:50, sc7383, Santa Cruz) and Ki67 (1:50, MA5-14520, Thermo Fisher Scientific) signals were used in this study and were amplified using TSA Fluorescein System (NEL701001KT, Perkin Elmer, Waltham, MA, USA), followed by secondary antibody incubation (1:300 donkey anti mouse-biotinylated IgG-B sc-2098 Santa Cruz, donkey anti rabbit-biotinylated IgG-B sc-2089 Santa Cruz). pERK (1:400, 4370, Cell Signaling) signal was obtained via DAB detection method. Nuclei were stained using Harris counterstaining and mounted using DPX. Pictures were taken with a Zeiss AxioImager Z1 Microscope and AxioVision SE64 software. Images were merged and/or quantified using ImageJ software (NIH) and QuPath (quantitative pathology & bioimage analysis) [13].

### Western blotting

AMG-treated fibroblasts as well as unwounded and MG-treated keratinocytes were used to evaluate changes in protein levels upon treatment (untreated cells were used as controls). Samples were processed as [14]. Membranes were then incubated overnight at 4 °C with primary antibodies: rabbit α-Smooth Muscle actin (1:800, ab15734, Abcam), mouse p-ERK (1:1000, sc-7383, Santa Cruz), rabbit ERK (1:1000, sc-94, Santa Cruz), mouse Tubulin-alpha (1:1000, T5168, Sigma Aldrich). Analyses were performed using the ChemiDoc XRS + detection system (BioRad, Temse, Belgium) in combination with chemiluminescent HRP substrates SuperSignal West Pico PLUS and SuperSignal West Femto (Thermo Fisher Scientific). Relative densitometry was obtained normalizing all samples to the housekeeping Tubulin-alpha, using both the QuantityOne software and ImageLab software (BioRad).

### Growth factors antibody array

Simultaneous detection of several growth factors within AMG was assessed using Mouse Growth Factor Antibody Array Membrane containing 30 targets, following manufacturer instructions (Cat No. AAM-GF-3-2, RayBiotech). Relative densitometry was obtained using the QuantityOne software (BioRad) and normalizing all signals to the positive controls signals of each membrane.

### Mass spectrometry analysis

The protein content of the extract was estimated based on Bradford protein quantification method. Subsequently, 300 µg of total protein extract from AMG was loaded into a gel and stained with Coomassie blue. The lane part below 30 kDa was cut with a sterile scalpel in tiny cubes (1 × 1 mm). The resulting gel pieces were extracted based on the protocol of Shevchenko et al. for the in-gel reduction, alkylation and destaining [15]. Peptide extracts were cleaned using C18 solid phase extraction according to the manufacturer (Pierce™ C18 Spin Columns, Thermo Fisher Scientific, Gent, Belgium) and dissolved in 5% ACN, 0.1% formic acid. Peptides from the candidate proteins were ordered from Thermo Fisher Scientific (UK) (PEPotec Grade 1). The UPLC–MS/MS analysis was performed with an Ultimate 3000 UPLC system (Dionex, Thermo Scientific) equipped with an EasySpray C18 column (3 μm, 75 μm × 15 cm, Thermo Scientific) using a gradient of 5% to 20% ACN in 0.1% formic acid (FA) for 10 min followed by a gradient of 10% to 35% ACN in 0.1% FA for 4 min and a final gradient from 35% to 95% ACN in 0.1% FA for 2.5 min and a Q Exactive Orbitrap mass spectrometer (Thermo Scientific, USA) was used for the DDA analysis of the peptide library and the PRM. The most suitable peptide per protein was chosen to set the PRM analysis. A dilution series of the library peptides was run at 120000 pg, 12000 pg, 1200 pg, 600 pg and 300 pg to correlate the peak area to concentrations (see library peptide information in supplementary information). PRM analysis was performed in Skyline 4.2. PRM runs were loaded as raw files with the following settings: MS1 orbitrap detection 70 000 resolution MS2 orbitrap detection 20 000 resolution. Only peptides showing 2 accurate transitions were accepted. All the AMG-derived proteins identified via MSMS are reported in the Supplementary Table S4.

### Matrix metalloproteinases (MMPs) enzymatic activity

MMPs activity was detected using MMP activity Assay Kit (Cat No. ab112146 Abcam), following manufacturer instructions. Conditioned serum-free media was added to  scratched and AMG-treated fibroblasts as well as from MG-treated keratinocytes and was collected 24 hours after to perform the analysis.

### Transwell migration assay

Transwell multiple well plates with permeable polycarbonate membrane inserts (Cat No. 3422, Thermo Fisher Scientific) were used for this study. 10^5^ cells were plated on the upper layer of the transwell in 100 μL of cell culture media. 600 μL of unprocessed AMG (1:10 diluted in Growth medium) or 600 μL of AMG soluble fraction (1:10 diluted) were added in the lower part of the chamber. A well containing 10^5^ cell and 600 μL of complete media was used as a control. After incubation time, the transwell inserts were removed from the chemo-attractant factors, cleaned from remaining media and not migrated cells using a cotton-tipped applicator and fixed with 70% ethanol. Afterward, membranes were left to dry and stained with 0,1% of crystal violet. Lastly, membranes were detached from the inserts, deposited on slides and mounted using DPX. Picture of the membranes were taken with Axiovert 40 CFL connected with Axiocam MRc5 (Zeiss) and analyzed using ImageJ software.

### RNA extraction and gene expression analysis

Total RNA was purified using PureLink® RNA Mini Kit (Cat No. 12183018 A, Life Technologies™) according to the manufacturer’s instructions. cDNA was generated using 0.5 μg of RNA and obtained by the Superscript III Reverse Transcriptase First-Strand Synthesis SuperMix (Invitrogen). Quantitative real-time PCRs (qRT-PCR) were performed using the Platinum® SYBR® Green qRT-PCR SuperMix-UDG (Cat No. 11733038, Thermo Fisher Scientific) on a ViiA™ 7 Real-Time PCR System with 384-well plate (Cat No. 4453536, Applied Biosystems). Gene expression values were normalized based on the *Gapdh*, *Rpl13a and*, *B actin* for mouse primary fibroblasts and *GAPDH, RPL13A, and B ACTIN* housekeeping genes for human keratinocytes. All primers that were used were purchased from IDT technologies, Leuven, Belgium and are reported in Table S5.

### RNA sequencing and bioinformatics analyses

RNA samples were quantified with Nanodrop 1000 spectrophotometer (Thermo Fisher Scientific) and RNA integrity was evaluated using Bioanalyzer (Agilent 2100) combined with Agilent RNA 6000 Nano Kit (Ca No. 5067-1511). RNA samples were then processed by the Genomics Core Leuven (Belgium). Library preparation was performed with the Illumina TruSeq Stranded mRNA Sample Preparation Kit (48 samples). Libraries were sequenced on the Illumina HiSeq4000 sequencing system. 50 bp single-end reads were generated and an average of 20 million reads were obtained. Mapping was performed with TopHat v2.0.13 against the mouse genome mm10. Quantification of reads per gene was performed with HT-Seq count v0.5.3p3. Count-based differential expression analysis was done with R-based Bioconductor package DESeq. Data are available as a GEO dataset under accession no. GSE123829. A list of differentially expressed genes (DEGs) obtained from our expanded cohort of samples (N = 3) were selected at an adjust *p* value < 0.05 and used to perform enrichment analysis through Gene Ontology (GO) via Panther classification system (Table S2), as well as used to build the PPI network representing the WH process by using the public PPI repository STRING.

### N-of-1 pathway MixEnrich single-subject analysis (SSAs)

Using the *N-of-1 Pathway* MixEnrich analysis [16], we identified DEGs without the requirement of large cohorts or replicates by directly analyzing paired samples (AMG-treated vs untreated cells) drawn from the same animal upon different AMG treatment time points (1 h, 5 h, 12 h, and 24 h). All samples have been first normalized by using NOIseq [17, 18]. Next, for each transcriptome sample we computed the absolute value of log-transformed fold change *|log*_*2*_*FC|* as *|log*_*2*_*(U/T)|*, where *U* and *T* are the gene expression level in the untreated and AMG-treated condition, respectively. *N-of-1 Pathway* MixEnrich identifies dysregulated pathways with upregulated and downregulated genes (bidirectional dysregulation), which are ubiquitous in biological systems by first clustering genes into upregulated, downregulated and unaltered genes. Subsequently, MixEnrich identifies pathways enriched with upregulated and/or downregulated transcripts using a Fisher’s Exact Test (FET). Here, for each AMG time of treatment, the enrichment test detects only pathways with a significantly higher proportion of dysregulated genes with respect to the background. In this way, the approach is more robust in the presence of background noise (i.e., a large number of dysregulated genes unrelated to the phenotype). Since different pathways may not be independent due to overlapping genes between them, the FET *p* values obtained are adjusted for multiple hypothesis testing using Benjamini and Yekutieli approach [16].

### Network construction

The PPI network was constructed by using as seed nodes the protein codified by the DEGs resulting from SSAs and linking them using PPIs extracted from STRING v.10.5 [19]. According to our previous works [20, 21], we retained only the most reliable PPIs by considering only database or experimental evidence and STRING confidence score >700. The constructed network is also a weighted network where the edge weights correspond to the STRING confidence score associated with the PPI (edge).

### Hub nodes

We identified network hubs by retaining the top 10% of the highest degree nodes. This threshold was suggested by other studies [20] and allowed us to identify nodes having key roles in the network and therefore in the AMG treatment process. In fact, several studies demonstrated that hubs likely correspond to network nodes playing an important role in the system represented [22, 23].

### Network clustering

Topological clusters in PPI networks are likely to correspond to specific biological functions or biological processes [24], We therefore performed the clustering of our network followed by biological process enrichment of each cluster to detect the major biological processes represented in the network. The clustering was performed using the Cytoscape [25] plugin ClusterONE [26] since it considers network weights (i.e., STRING confidence scores), allows cluster overlapping and provides a *p* value for each cluster identified [26]. To distinguish significant clusters from non statistically significant ones, authors suggest a *p* value threshold of 0.05 [26] (Table S3).

### Hub and clusters enrichment analyses

We performed the enrichment of a group of genes, such as hubs or genes belonging to a specific cluster, using the Cytoscape plugin ClueGO [27]. ClueGO allows to automatically load the gene list of interest, perform the enrichment analysis using a source of knowledge (e.g., Gene Ontology Biological Processes (GO-BPs)), and to extract a list of significant terms and a network visualization of the results. We considered enriched all the GO-BPs whose *p* value was smaller than 0.05.

### ChIP-sequencing analysis

Publicly available ChIP-seq data [28] were processed using the pipeline from the Kundaje lab (Version 0.3.3). Reads were aligned to reference genome (mm10) using Bowtie2 (v2.2.6) using the ‘--local’ parameter. Single-end reads that aligned to the genome with mapping quality ≥ 30 were kept as usable reads (reads aligned to the mitochondrial genome were removed) using SAMtools (v1.2). PCR duplicates were removed using Picard’s MarkDuplicates (Picard v1.126). Coverage was calculated using bamCoverage function with binsize = 1 bp and normalized using RPKM.

### In vivo wound healing assay

Twenty 12 week-old female C57BL/6 mice were used in this study. Specifically, excisional wounds were made in the dorsal skin of four groups of C57BL/6 mice (*N* = 5) under ketamine/xylazine anesthesia with a 5-mm biopsy punch. [29, 30]. Silicon rings were sutured around the wounds to limit wound closure caused by skin contraction. Wounds were then topically treated with thirty microliters of AMG (diluted in DMSO, 1:3), obtained from the 5-mm skin biopsy following the Rigenera® protocol [5]. A second group was topically treated with AMG supplemented with the specific MEK inhibitor—trametinib (0.2 mg, Cat No. HY-10999, MCE). Trametinib was dissolved in dimethylsulfoxide (DMSO) to a concentration of 20 mg/ml and 10 μL of the mixture (corresponding to 0.2 mg) was added topically together with the AMG [31]. A third group was topically treated with the MEK inhibitor—trametinib (0.2 mg, Cat No. HY-10999, MCE) (1:3 in PBS). The control group was treated with PBS diluted 1:3 with DMSO as vehicle. Wounds were covered with Tegaderm™ dressing (3 M, Maplewood, USA) to protect the wound area and to prevent the area from drying out. Every other day (day 2, 4, 6, and 8), digital images were taken using a Canon EOS 5D Mark II camera, treatments were re-applied topically to the wound site and dressings were renewed. Changes in % of wound closure were calculated by comparing the healed wound area of specific days with the original wound area over the time, as shown in [32]. The analyses were performed using ImageJ software (NIH, Baltimore, Maryland). Animals were sacrificed at day 8 after wounding and wound skin fractions were collected for further paraffin embedding or gene expression analyses. Immunohistochemistry and immunofluorescence staining were performed on 7 μm microtome sections. All the experiments performed were approved by the Ethics Committee at KU Leuven University under the ethical approval codes P056/2017 and P036/2018).

### Histological staining

Paraffin sections were heated at 57 °C for 60 min, deparaffinized and rehydrated. Sirius Red solution was prepared by mixing 0.2 g of Direct Red 80 (Sigma-Aldrich) with saturated aqueous solution of picric acid (prepared mixing 8 g of picric acid in 200 mL of distilled water). Samples were stained with Sirius Red solution for 90 min. Slides were then washed with HCl 0.01 N for 2 min and dehydrated with ethanol 70% for 45 s and twice in ethanol 100% for 5 min. The samples were cleared in xylol for 5 min (twice), mounted with DPX and left on a slide heater overnight. Pictures were taken with an Axiovert 200 M motorized inverted microscope (Zeiss). The amount of organized collagen was revealed with polarized light microscopy on the same cross-sections. Collagen quantification was determined using ImageJ software (NIH). Hematoxylin and eosin staining was performed as in [33].

### Statistical analysis

Statistical analysis of RNA sequencing data were performed using Bioconductor package DESeq. Reported *p* values were adjusted for multiple testing with the Benjamini–Hochberg procedure, which controls false discovery rate (FDR). GraphPad Prism 6 was used to visualize the data and GraphPad Prism software (San Diego, CA, USA) was used to analyze all the experiments in this study. Significant differences were determined by one-way analysis of variance ANOVA (multiple groups) and multiple unpaired *t* test (two groups). Data are presented as mean ± standard error of mean (SEM) or as fold change.

## Results

### Wound-induced transcriptional network upon AMG treatment

Fibroblasts have a critical role during the process of WH due to their ability to migrate from the dermis to the wound site in response to the cytokine/growth factor gradients [34, 35]. As a first step to better understanding the molecular mechanisms driving the AMG effects, we set out to perform a broad transcriptome analysis of AMG-treated primary fibroblasts subject to in vitro scratch closure assay. Cells were treated with AMG for 1 h, 5 h, 12 h, and 24 h and were collected together with untreated wounded fibroblasts as a control for RNA-seq analysis (GSE123829). Using the N-of-1 Pathway MixEnrich analysis [16], which allows the identification of DEGs without the requirement of large cohorts or replicates, we identified DEGs (Fig. S1A). The analysis showed that both AMG and the duration of its exposure to the fibroblasts influence the transcriptome profile compared with untreated cells (Fig. S1B). Interestingly, 5 h AMG-treated cells showed enrichment of 40 gene ontology (GO) WH-related signaling pathways such as regulation of cell motility, inflammatory response, and ERK/MAPK cascade. In contrast, 12 and 24 h AMG-treated cells showed enrichment of 482 and 581 GO pathways, respectively, suggesting accumulation of non-direct responses related to the treatment (Table S1; Fig. S1C, D). We concluded that 5 h of treatment most likely best represented the initial and direct AMG effects at transcriptome level. Therefore, we expanded our biological cohort to *N* = 3 by performing additional RNA-seq experiments at 5 h AMG treatment.

**Fig. 1:**
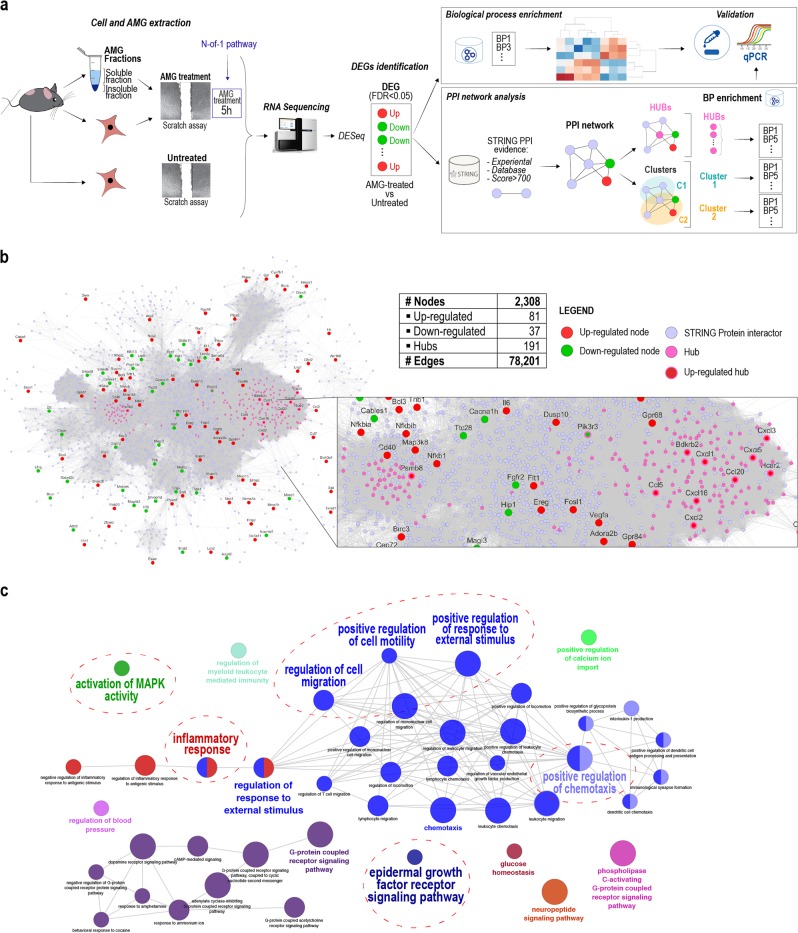
Comparative transcriptome analysis of primary murine fibroblasts upon AMG treatment. **a** Schematic representation of the strategy followed to reveal AMG-dependent mechanisms on fibroblasts. Transcriptome analysis via RNA-seq of AMG-treated and untreated murine primary fibroblasts led to the identification of AMG-related DEGs. Those genes were then used to perform a double strategy analysis: (1) Enrichment analysis of biological processes through GO and (2) PPI network analysis to predict DEGs-associated proteins and signaling pathways involved in the AMG molecular mechanism. **b** The Protein–Protein Interaction Network (PPI) referred to the list of DEGs observed upon AMG treatment and obtained by the STRING repository. DEGs are represented as red and green nodes of the network, based on their upregulated or downregulated expression, respectively. Hub nodes are presented as purple nodes shown in an enlarged view. **c** Hub related GO enrichment analysis with highlighted biological process playing essential roles in the WH process

Subsequently, DEGs between AMG-treated and untreated cells were employed to conduct a conventional transcriptome analysis and PPI network analysis, both followed by enrichment analyses (Fig. 1a). Conventional transcriptome analysis showed 203 DEGs (Fig. S1E; Table S2) and 548 significantly enriched biological processes between AMG treated and untreated samples using GO terms (GO-BP) (Table S2) [36]. GO-BP identified biological processes and signaling pathways involved in WH such as cell migration, immune response, angiogenesis, and the MAPK cascade (Fig. S1F, H; Table S2). Moreover, several genes such as those encoding cytokines and MMPs were differentially expressed (Table S2; Fig. S1G, I), suggesting that AMG treatment rapidly induces changes in expression of genes associated with a successful WH process.

Based on the identified DEGs and the public PPI repository STRING [19] (see materials and methods—Network construction), we constructed a PPI network consisting of 2308 protein nodes and 78,201 PPI edges (Fig. 1b; Table S3). Through topological network analysis, we derived 191 key hub nodes (see material and methods—Hub nodes; Table S3) and 49 significant network clusters (see material and methods—clusters; Table S3, Fig. S2A). Hub-related GO enrichment analysis highlighted several biological processes playing essential roles in the AMG-mediated WH (Fig. 1c). Interestingly, within the top 10 most significant clusters after enrichment analysis, we found four clusters (Cluster 1, 2, 6, and 7; Fig. S2A) to be associated with WH and tissue regeneration processes (Fig. S2B–E), in line with the findings of our transcriptome analysis.

Altogether, we conclude that AMG treatment enhances the healing process by exerting fibroblasts activation and by stimulating the transcription of genes, which are crucial for WH-associated biological processes.

### AMG treatment increases cell migration and accelerates in vitro scratch closure

**Fig. 2:**
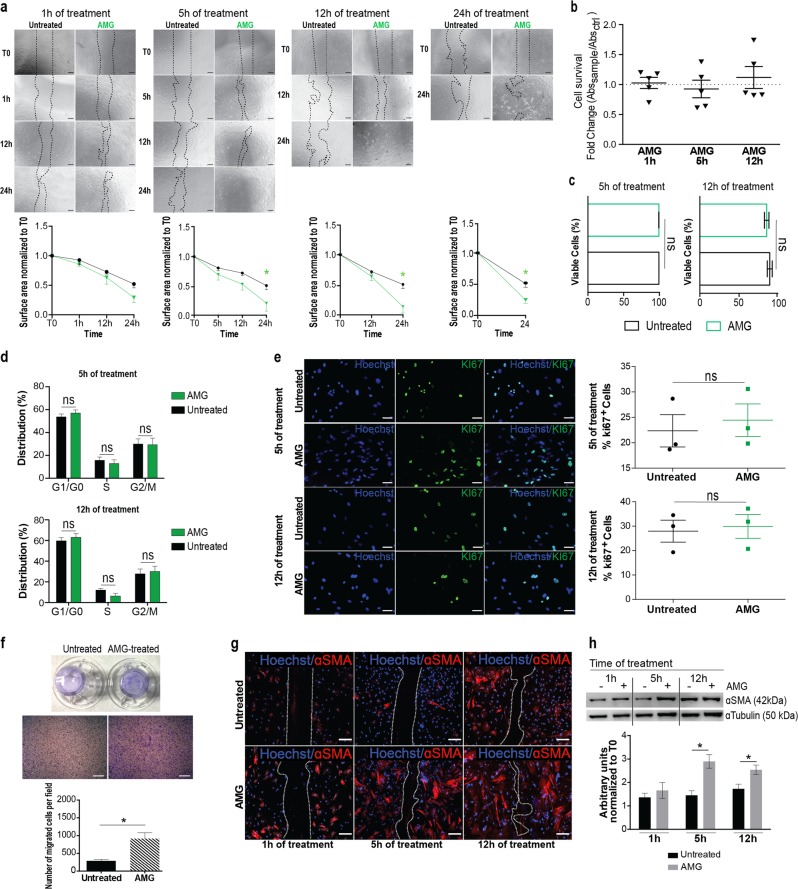
AMG treatment increases migration capacity of murine primary fibroblasts. **a** Representative images (up) and quantification (down) of AMG-treated and untreated fibroblasts in a scratch wound assay. AMG was applied for different time periods (1, 5, 12, and 24 h). Cells treated with AMG for 5 and 12 h exhibited the quickest closure of the scratch wounds with a closure percentage of 77% ± 11% and 84% ± 8%, respectively at 24 h compared with untreated cells (48% ± 26%). Quantification averages of *N* = 4 for each time of treatment are reported below. Data are presented as mean ± SEM (standard error of the mean). Significant differences vs control are calculated multiple unpaired *t* test and indicated as *P < 0.05. Scale bar = 200 μm. **b** XTT assays were used to measure cell survival upon AMG-based treatment. Absorbance values (read at 450 nm) were normalized to the control group (untreated) and expressed as a fold change (*N* = 5). **c** Cell viability was evaluated by flow cytometry using the fixable viability stain 660. Percentage of viable cells upon 5 and 12 h of AMG treatment are reported. Statistical analyses were performed using two-tailed unpaired *t* test. No significant differences were found. (*N* = 3). **d** Cell cycle FACS analyses of AMG-treated growing fibroblasts upon 5 and 12 h of AMG treatment. EdU and PI staining was performed to detect cell cycle changes. Quantification average of *N* = 4 is reported. Data are presented as mean ± SEM. Multiple unpaired *t* test was used to perform statistical analysis. No significant differences were found between AMG-treated and untreated cells. **e** Representative immunofluorescence showing Ki67 levels from 5 and 12 h AMG-treated primary growing fibroblasts. Nuclei were counterstained with Hoechst 33342. ns Not significant, (*N* = 3). Scale bar = 100 μm. **f** Macro- and microscopic observation of untreated and AMG-treated transwell chambers. Migrated cells were stained with crystal violet (0.1%), observed under a light microscope and analyzed using ImageJ software. Data are presented as mean ± SEM. *P* values were calculated using student *t* test (*N* = 4) *P < 0.05. Scale bar = 100 μm. **g** Representative immunofluorescence for ɑ-SMA protein localization (in red). Nuclei were stained with Hoechst (blue). Scale bar = 100 μm. **h** Representative western blotting showing ɑ-SMA protein level extracted from AMG-treated (+) or untreated (−) wounded murine fibroblasts. Quantification of band areas was determined by densitometry software and normalized to ɑ-Tubulin loading control. Significant differences were calculated using multiple unpaired *t* test (*N* = 3) and indicated as *P < 0.05

Murine primary adult fibroblasts were isolated from tail tips and cultured to perform scratch closure assays upon the exposure to AMG extract for 1, 5, 12, and 24 h. Cells treated with AMG for 5 and 12 h exhibited the fastest significant closure compared with untreated cells (Fig. 2a), suggesting that AMG treatment might have a positive effect on cell proliferation and/or cell viability and/or cell migration. Therefore, we examined AMG-dependent effects on 60–70% confluent primary fibroblasts by performing cell survival/viability and cell cycle assessment. Interestingly, cell survival (Fig. 2b, c), cell cycle (Figs. 2d, S3A) and the evaluation of Ki67^+^ cells (Fig. 2e) were unaffected by AMG treatment, excluding a direct effect on fibroblast cell proliferation and viability. Remarkably, AMG-treated cells showed increased cell migration (Fig. 2f) and enhanced expression of the myofibroblast marker alpha-Smooth Muscle Actin (*ɑ-SMA*) by immunostaining and western blotting analysis (Figs. 2g, h, S3B).

In conclusion, the results of these functional assays indicate that AMG treatment increases cell migration and accelerates in vitro fibroblast scratch closure with no significant effect on fibroblast proliferation and viability.

### Soluble AMG fraction accelerates scratch wound closure compared with unprocessed micrograft

Although studies have suggested the beneficial role of mesenchymal stem cells (MSCs) within the AMG tissue extract [1, 4], an AMG fractioning has not yet been carried out to address this issue. Using a polyethylene glycol (PEG) membrane enrichment method, we separated the soluble fraction (SF-AMG) containing essential growth factors from the insoluble fraction (IF-AMG) containing cellular membrane vesicles. Next, scratched fibroblasts were treated with unprocessed AMG or SF-AMG or IF-AMG fraction for 5 h.

**Fig. 3:**
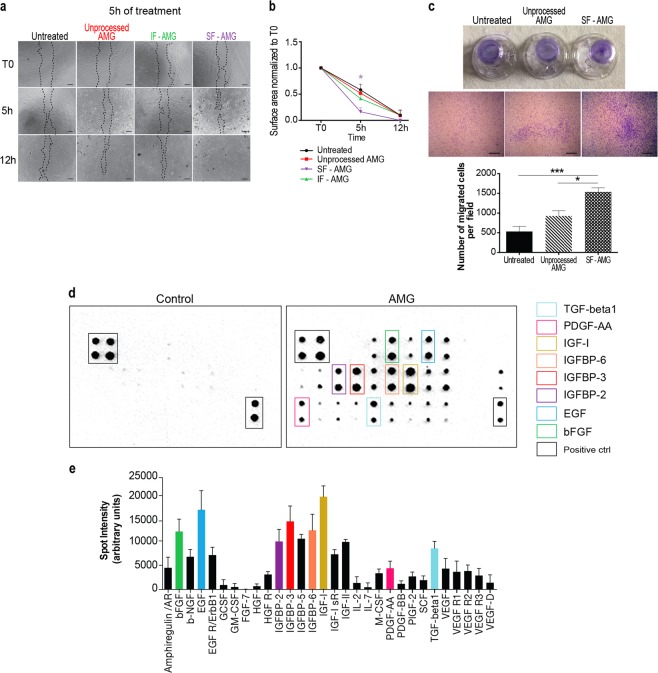
Characterization of AMG molecular fractions. **a** Representative image of scratch assay on AMG-treated (5 h) murine primary fibroblasts (unprocessed, insoluble—IF and soluble—SF AMG fractions). Scratched untreated cells were used as a control. At 5 h after treatment, untreated, or treated samples with unprocessed or IF-AMG fraction showed ~50% and 60% of wound closure with no significant differences between conditions. In contrast, SF-AMG fraction treatment showed 84% of wound closure with complete closure 12 h after in vitro wounding. Scale bar = 200 μm. **b** Quantification average for each time of treatment. Data are presented as mean ± SEM. Differences are calculated with one-way ANOVA (*N* = 3) and indicated as *P < 0.05. **c** Observation of transwell chambers after stimulation with the AMG treatment. Migrated cells were stained with crystal violet 0.1%, observed under a light microscope and analyzed using ImageJ software. Data were presented as mean ± SEM. Differences are calculated using one-way ANOVA followed by Tukey test (*N* = 4) and indicated as *P < 0.05; ***P < 0.005. Scale bar = 100 μm. **d** Representative images of mouse antibody array membrane showing presence of several growth factors within the AMG soluble fraction (right panel) compared with the PBS vehicle (left panel) (*N* = 4). **e** Analysis of the increase in spot intensity (in arbitrary units) is shown in the graph

SF-AMG-treated cells showed complete closure of the scratch after 12 h, indicating that SF-AMG significantly accelerates cell migration and, in turn, scratch closure (Fig. 3a, b, c).

Interestingly, members of the IGF family, especially IGF-I as well as EGF, bFGF, and TGF- β1, were detected within the solution using antibody array membranes. (Fig. 3d, e). Through Liquid Chromatography Mass Spectrometry (LC-MS) analysis, we assessed absolute quantification of AMG-derived IGF-I and bFGF (Figs. S4A, B; S5A, B). Particularly, we estimated an absolute concentration of 4 ng/mL for IGF-I and 0,5 ng/mL for bFGF in the AMG solution.

These findings indicate that AMG extract and SF-AMG are expecially enriched with pro-motility growth factors, which explain the increased cell migration induced by the AMG treatment.

### MMPs trigger AMG-mediated cell migration capacity

**Fig. 4:**
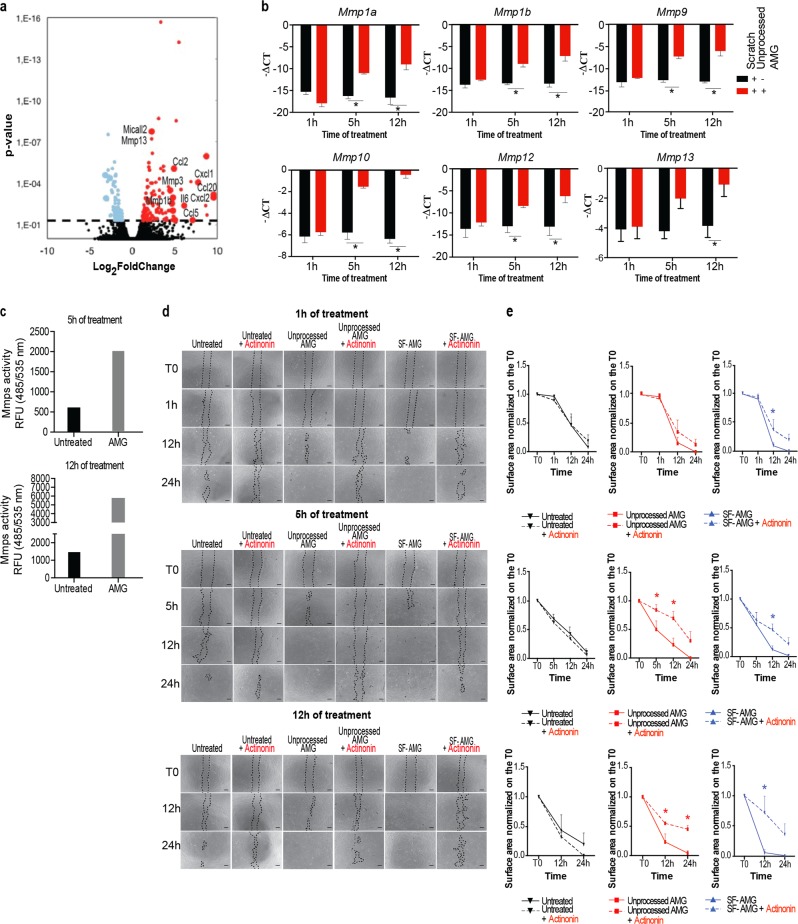
AMG-based treatment triggers matrix metalloproteinase expression and enzymatic activity in murine primary fibroblasts. **a** Volcano plot showing up- (red dots) and down-regulated (light blue dots) genes altered by AMG treatment (5 h of stimulation). Gene values are reported as a Log_2_FoldChange (*p* value < 0.05). **b** MMP gene expression evaluated by RT-qPCR. Expression values are expressed as a -∆CT normalized on the expression of *Gapdh*, *B-actin,* and *Rpl13a* housekeeping genes (*N* = 3). Differences are calculated with multiple unpaired *t* test (*N* = 3) and indicated as *P < 0.05. **c** Enzymatic activity of MMP members present in cell supernatant was fluorometrically detected. Data are presented as Relative Fluorescence Units (RFU). Signals were evaluated 30 min after starting the reaction using a microplate reader with a filter set of Ex/Em = 485/535. The fluorescence signal obtained from each sample was normalized on the substrate control. Groups of wounded cells that did not receive any treatment were used as a control. **d** Representative images of scratch wound assays on monolayers of murine primary fibroblasts. AMG was applied on wounded cells with different conditions (unprocessed AMG and its soluble fraction) and for different time periods (1, 5, and 12 h). Each condition was also incubated with the MMPs inhibitor—Actinonin—20 μM for the same time periods of AMG treatment. Images were taken at the beginning (T0) and at regular intervals until wound closure was achieved. Scale bar = 200 μm. **e** Quantifications of AMG-treated cells with or without Actinonin are reported. Data are presented as mean ± SEM. Differences were calculated with multiple unpaired *t* test (*N* = 3) and indicated as *P < 0.05

We hypothesized that soluble AMG-derived growth factors are responsible for the activation of the pro-motility transcriptional program previously identified by PPI and GO enrichment analyses (Figs. 1c, S1F, G, and S2C). By RNA-seq analysis we selected 203 DEGs as key regulators involved in AMG induced process, (log_2_FC >1, FDR <0.05) (Fig. 4a). Among those, we identified several upregulated members of the MMP family in the AMG-treated conditions. These genes were then validated by qPCR confirming the induction of *Mmp1a-1b* (known as collagenase-1), *Mmp9* (known as gelatinase B), *Mmp10* (stromelysin-2), *Mmp12* (metalloelastase) and *Mmp13*, expression upon AMG treatment (Fig. 4b). Accordingly, the increased expression of MMP was functional as extracellular proteinase activity was enhanced in collected conditioned media obtained from AMG exposed fibroblasts (Fig. 4c).

Furthermore, we tested whether MMP were responsible for the higher cell motility by performing scratch closure assay upon AMG or SF-AMG treatment in the presence of the MMP-specific inhibitor Actinonin (Fig. S6A). Of note, inhibition of MMP activity resulted in a significant delay in scratch closure, demonstrating that MMP activity plays a deterministic role in AMG-dependent cell migration (Fig. 4d, e). As MMP expression in fibroblasts has been reported to be partially regulated by the activated protein 1 (AP-1) transcription factor activity [37] we verified that members of the AP-1 family, such as *Fosl1*, *Fosl2*, *cJun*, and *Junb*, were induced upon AMG treatment (Fig. S6B). In addition, using publicly available ChIP-seq data, we identified Fra1, a member of AP1 complex, as a candidate protein for the binding to the regulatory regions of MMPs (Fig. S6C).

Taken together, our data demonstrate that AMG treatment increases MMPs expression and their extracellular activity, which are essential in mediating enhanced cell migration.

### AMG treatment induces ERK–dependent MMP expression and cell migration

**Fig. 5:**
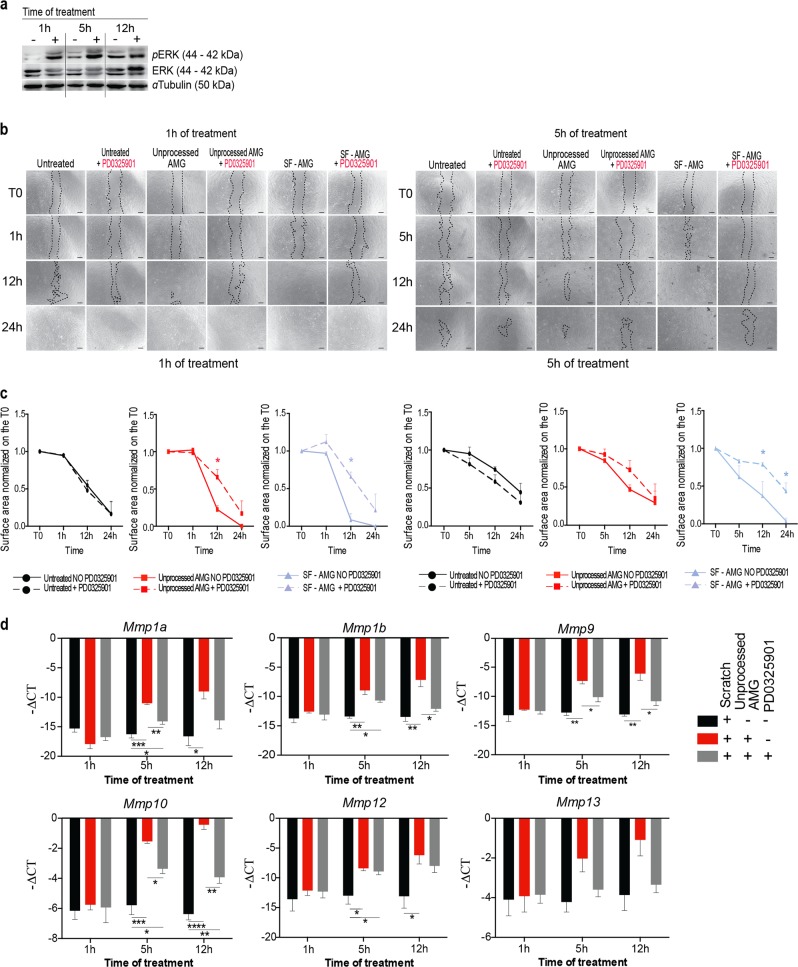
ERK/MAPK signaling pathway is specifically activated by AMG treatment. **a** Representative western blotting out of three biological replicates showing protein level of phosphorylated and total ERK1/ERK2 from wounded fibroblasts upon AMG treatment. **b** Representative images of scratch wound assays. Unprocessed AMG and its soluble fraction (SF) were applied on wounded cells. Each condition was also incubated with the MEK inhibitor—PD0325901—1 μM for the same time periods of the AMG treatment. Wounded cells did not receive any treatment and were used as a control. Scale bar = 200 μm. **c** Quantifications of AMG-treated cells with or without PD0325901 for each time of treatment are reported. Data are presented as mean ± SEM. Significant differences vs control were calculated with multiple unpaired *t* test (*N* = 3) and indicated as *P < 0.05. **d** MMP gene expression evaluated by RT-qPCR upon AMG treatment and MEK inhibitor PD0325901 exposition. Expression values are expressed as a -∆CT normalized on the expression of *Gapdh*, *B-actin,* and *Rpl13a* housekeeping genes. Differences are calculated with one-way ANOVA (*N* = 3) and indicated as *P < 0.05; **P < 0.01; ***P < 0.005; ****P < 0.001

During WH, tissue regeneration is achieved by activation of crucial signaling pathways. GO and Network-based analysis performed on fibroblasts upon 5 h of AMG treatment showed enrichment of the MAPK cascade and extracellular signal-regulated kinase (ERK) (Figs. S1H, S2D). Accordingly, AMG treatment showed the upregulation of several ERK pathway target genes (Fig. S1I). Interestingly, SF-AMG contains the growth factors IGF-I, EGF, and bFGF, which are known to directly activate this intracellular pathway [38]. Therefore, we determined the levels of ERK phosphorylation (pERK) in scratched fibroblasts upon AMG treatment. Increased pERK levels in AMG-treated cells (Fig. 5a) demonstrated that AMG promotes ERK activation in treated fibroblasts. AMG-treated fibroblasts were scratched in the presence of the mitogen-activated protein kinase (MEK) inhibitor. ERK inhibition was confirmed by western blotting analysis (Fig. S7A), and significantly decreased cell migration and delayed scratch closure in both unprocessed AMG and SF-AMG conditions (Fig. 5b, c). Furthermore, activation of the ERK signaling pathway has already been reported to correlate with increased expression of different members of the MMP family members through AP-1 activity [39]. Accordingly, we showed that ERK inhibition in AMG-treated fibroblasts significantly reduced expression levels of *Mmp1a*-*1b*-*9-* and *10* in AMG-treated fibroblasts (Fig. 5d). Surprisingly, levels of *Mmp12* remained unchanged upon MEK inhibition, indicating involvement of other signaling pathways in the *Mmp12* regulation (Fig. 5d).

Collectively, our findings connect the AMG-mediated effects on cell migration through an ERK-dependent MMP induction, which might be induced by growth factors promoting cell motility.

### Micrograft (MG) treatment enhances cell migration of keratinocytes through ERK signaling pathway activation

**Fig. 6:**
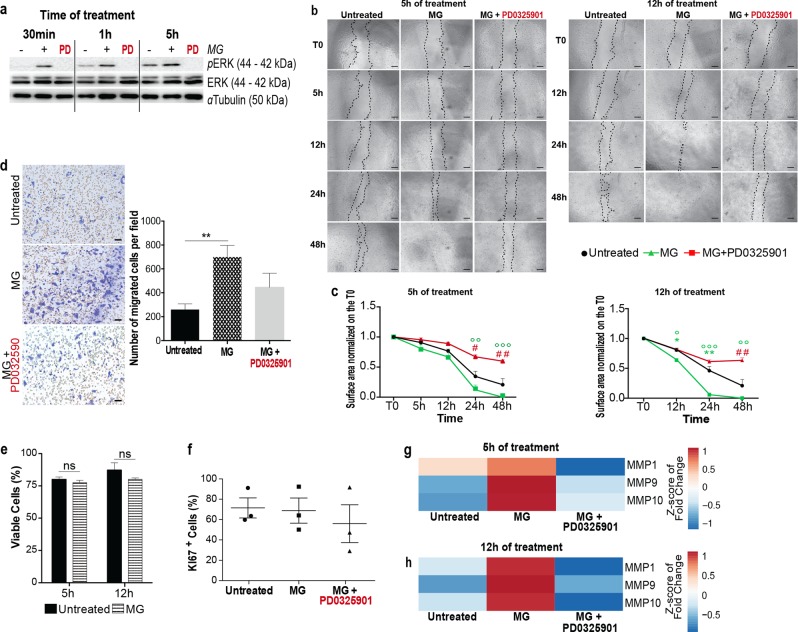
MG treatment promotes matrix remodeling through accelerated migration and enhanced MMP activity in keratinocytes. **a** Representative western blotting showing protein level of phosphorylated and total ERK1/ERK2 obtained from unwounded human keratinocytes upon 30 min, 1 and 5 h of MG treatment and in which we induced ERK inhibition (using the MEK inhibitor PD0325901—1 μM). **b** Representative images of MG-treated keratinocytes in a scratch wound assay. MG was applied for different time periods (5 and 12 h). Images were taken at the beginning (T0) and at regular intervals until closure was achieved. Each condition was also incubated with the MEK inhibitor—PD0325901—1 μM for the same time periods of the MG treatment. Wounded cells did not receive any treatment and were used as a control. Scale bar = 200 μm. **c** Quantifications of each time of treatment are reported in the graphs. Data are presented as mean ± SEM (standard error of the mean). Significant differences between control vs AMG are calculated with one-way ANOVA and indicated as *P < 0.05; **P < 0.01. Significant differences between AMG vs AMG + PD0325901 are calculated with one-way ANOVA and indicated as °*P* < 0.05; °°*P* < 0.01; °°°*P* < 0.005. Significant differences between control vs AMG + PD0325901 are calculated with one-way ANOVA and indicated as #*P* < 0.05; ##*P* < 0.01. (*N* = 3). **d** Observation of transwell chambers. Migrated cells were stained with crystal violet 0.1%, observed under a light microscope and analyzed using ImageJ software. Data were presented as mean ± SEM. Differences are calculated using one-way ANOVA (*N* = 4) and indicated as **P < 0.01. Scale bar = 100 μm. **e** Keratinocytes viability upon MG treatment was evaluated by flow cytometry using the fixable viability stain 660. Percentage of viable cells upon 5 and 12 h of AMG treatment are reported. Statistical analyses were performed using two-tailed unpaired *t* test. No significant differences were found between AMG-treated and untreated cells. (*N* = 3). **f** Ki67 staining analyses on AMG-treated keratinocytes (5 h and 12 h). Together with MG, the MEK inhibitor—PD0325901—was applied on human keratinocytes to assess ERK signaling involvement in cell proliferation. Untreated cells were used as controls. No differences in percentage of Ki67^+^ cells were evaluated in all the conditions under study. Data are presented as mean ± SEM. One-way ANOVA was used to perform statistical analysis (*N* = 3). **g** MMP gene expression evaluated by RT-qPCR in vehicle and 5 h AMG-treated in the presence or absence of PD0325901 1 μM. Expression values are expressed as a z-score of the average fold change (FC) normalized on the expression of *GAPDH*, *B-ACTIN*, and *RPL13A* housekeeping genes (*N* = 3). **h** MMP gene expression evaluated by RT-qPCR in vehicle and 12 h AMG-treated in the presence or absence of PD0325901 1 μM. Expression values are expressed as a z-score of the average fold change (FC) normalized on the expression of *GAPDH*, *B-ACTIN*, and *RPL13A* housekeeping genes (*N* = 3)

Migration of keratinocyte is essential for successful WH [40]. To study the effect of AMG extract on an epithelial cell model, we performed scratch assay on HaCaT keratinocyte cell line [41]. Also in this case, the non autologous MG extract induced ERK activation (Fig. 6a) and a significant increased scratch closure capacity (Fig. 6b, c). Interestingly, MG-increased motility of epithelial cells was significantly reverted by the application of MEK inhibitor (Fig. 6b, c). In addition, transwell migration assay confirmed the enhancement of epithelial cell migration upon MG treatment (Fig. 6d). We reported no differences in cell viability or cell proliferation in MG-treated and untreated cells (Figs. 6e, f, S8A). Also in the epithelial cells model, the expression levels of MMPs were increased upon 5 h and 12 h of MG treatment (Fig. 6g, h). MMPs enzymatic activity assay confirmed their activation upon MG treatment that was reverted upon MEK inhibition (Fig. S8B).

Altogether these results suggest that MG treatment regulates important epithelial cellular functions through the ERK activation, which in turn leads to a faster healing process.

### ERK signaling pathway drives AMG-mediated WH

**Fig. 7:**
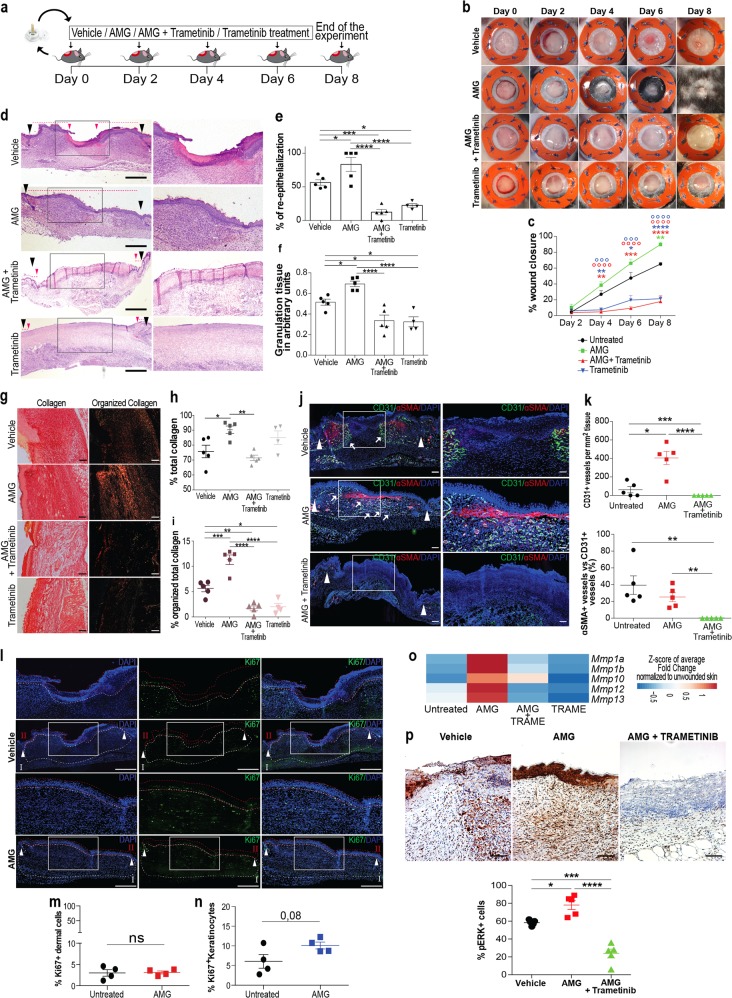
The in vivo healing potential of AMG through ERK signaling pathway. **a** Schematic representation of the in vivo WH study performed on C57BL/6 mice. Animals were topically treated with vehicle, AMG, and MEK inhibitor—trametinib (0.2 mg) on day 0, 2, 4, and 6. At the end of the experiment (day 8), skin wound samples were collected for further analyses. **b** Excisional wound-splinting assay showing the potential of AMG to improve wound closure in AMG-treated mice compared with the other conditions under study (vehicle, AMG + trametinib and trametinib). Moreover, two out of five AMG-treated animals showed hair surrounding the wounds. **c** Percentage of wound closure between the groups under study. **d** Representative hematoxylin and eosin (H&E) stained section on day 8 after wounding. Scale bar 500 μm. Arrowheads delimitate the wound area and 2x zoomed region of interest is reported on the right. Pink dotted lines and arrowheads delimitate the epithelial tongues. **e**, **f** Percentage (%) of re-epithelialization and granulation tissue formation expressed in arbitrary units (AU) among the evaluated groups. Re-epithelialization coverage in wounds treated with AMG reached 83% ± 10% compared with only 56% ± 4% in vehicle-treated mice. Percentage of GT formation of AMG-treated group was significantly higher (70% ± 2,7%) compared with the other conditions, suggesting increased fibroplasia in AMG-treated mice. **g** Representative Sirius Red (SR) stained section on day 8 after wounding. Scale bar 100 μm. **h**, **i** % of total collagen formation and % organized collagen on the total collagen was evaluated between the conditions. **j** Representative immunofluorescence of vehicle and AMG-treated wounds exposed or not to trametinib, showing presence of CD31 + and ɑ-SMA + vessels. Scale bar 200 μm. Arrowheads delimitate the wound area and 2x zoomed region of interest is reported on the right. Arrows indicate CD31 + vessels. **k** Number of CD31 + per mm^2^ tissue and ɑ-SMA coated CD31 + vessels were evaluated in the entire wound area. **l** Representative immunofluorescence of vehicle and AMG-treated wounds showing the presence of the proliferative marker Ki67. % of Ki67 + cells were evaluated in both dermal (White region—I) and epidermal (Red region—II) layers (*N* = 4). Scale bare 500 μm. Arrowheads delimitate the wound area and 2x zoomed region of interest is reported above the images. **m** No significant differences were found in the percentage of Ki67 + cells in the dermal layer. Two-tailed unpaired *t* test was used to perform statistical analysis. **n** Percentage of Ki67 + cells in the epidermal layer. Two-tailed unpaired *t* test was used to perform statistical analysis (*p* < 0,08). **o** MMP gene expression evaluated by RT-qPCR in vehicle and AMG-treated in presence or absence of trametinib. Expression values are expressed as a z-score of the average fold change (FC) normalized on the expression of *Gapdh*, *B-actin,* and *Rpl13a* housekeeping genes (*N* = 3). **p** Representative immunohistochemistry for pERK in vehicle and AMG-treated in presence or absence of trametinib. Scale bar 100 μm. Quantification of pERK^+^ cells (%) analyzed using QuPath software. All data are presented as mean ± SEM. Differences are calculated using one-way ANOVA (*N* = 5) and indicated as *P < 0.05; ***P < 0.005; ****P < 0.001

Given the power of AMG extract in promoting in vitro cell migration and scratch closure, we used the excisional WH mouse model to verify the AMG extract ability in promoting tissue repair in vivo [31]. Wounds comprising the entire dermis and epidermis were generated on the back of C57BL/6 mice and were splinted with silicone rings to limit wound closure by skin contraction. Subsequently, animals were topically and repeatedly treated with AMG obtained from autologous skin biopsies in the presence or absence of trametinib, a MEK inhibitor (Fig. 7a). The topical application of AMG significantly accelerates wound closure on days 4, 6, and 8 compared with vehicle-treated mice. Furthermore, topical administration of trametinib significantly delayed wound closure in all conditions, confirming the essential role of the ERK signaling pathway in AMG-mediated WH (Fig. 7b, c). Next, we assessed the AMG benefits on epidermal healing by considering the percentage of re-epithelialization. While none of vehicle-treated mice showed complete epithelial coverage at day 8 after injury, three out of the five AMG-treated mice exhibited complete re-epithelialization. Importantly, trametinib treatment significantly reduced re-epithelialization compared with vehicle and AMG-treated mice (Fig. 7d, e). Afterward, we evaluated granulation tissue (GT) formation, angiogenesis and the deposition and organization of new collagen fibers. Fibroplasia and angiogenesis have both been reported to be indispensable events needed for successful WH [42]. We demonstrated that GT formation was significantly higher in AMG-treated mice, suggesting increased fibroplasia (Fig. 7f). The amount of organized (red birefringent) and total collagen was significantly greater in AMG-treated wounds compared with the vehicle-treated ones. Importantly, trametinib reduced the amount of both polarized and total collagen upon AMG treatment (Fig. 7g, h, i). Furthermore, analysis of endothelial marker CD31 and αSMA revealed increased CD31^+^ vessel formation, indicating the potential of AMG to induce angiogenesis. However, as marked by low levels of αSMA, newly formed vessels showed to be immature at the time of the analysis (Fig. 7j, k). In addition, to assess the AMG effect on cell proliferation in vivo, wound areas were histologically evaluated for Ki67^+^ cells which were quantified in both dermal and epidermal layers (Fig. 7l). Although no significant differences in Ki67^+^ cells were found in dermal cells (Fig. 7m), AMG-treated wounds showed a tendency for an increased amount of Ki67^+^ cells in the epidermis (*p* value 0.08) (Fig. 7n).

Interestingly, levels of *Mmp1a, 1b, 10, 12*, and *Mmp13* were increased in AMG-treated mice. Treatment with trametinib reduced expression of all MMPs, suggesting a strong ERK-dependent MMPs gene regulation upon AMG treatment in vivo (Fig. 7o). In addition, phosphorylation of ERK was evaluated by immunohistochemistry, confirming the ERK activation by AMG treatment (Fig. 7p).

Altogether our results demonstrate the ability of AMG treatment to actively trigger the proliferative and remodeling phases of in vivo WH, leading to an acceleration of the entire process of tissue repair in an ERK-dependent manner.

## Discussion

Although the physiological healing of wounds is highly effective, it can be restricted by the extension of the affected area as well as by several other factors [43, 44]. Nowadays, the wound management field is impacted by excessive costs and detrimental physical/psychological side effects for patients. Therefore, AMG technology represents a low cost, accessible and clinically effective alternative to improve tissue regeneration.

AMG has been shown to provide positive clinical outcomes with significantly accelerated WH [1–5]. However, the molecular mechanisms connecting its beneficial outcomes with the WH process remain unknown. Herein, we demonstrate that AMG-induced transcriptional signature is enriched in genes related to WH-associated biological processes and, specifically, to signaling pathways driving cell migration, angiogenesis, and MAPK/ERK activation. Functionally, we reveal that AMG treatment promotes migration of fibroblasts and keratinocytes, contributing to accelerate the WH process. Importantly, we coupled this effect with an increased MMPs activity, which is sustained by the ERK signaling pathway. In accordance with our findings, topical application of MEK inhibitor—trametinib—impairs in vivo AMG beneficial effects. Our study results have enabled us to provide a framework for how AMG supports WH, opening avenues for further clinical advances.

Upon injury, the earliest event driving the WH response is characterized by gradients of cytokines/growth factors such as interleukin-6 (IL-6), EGF, PDGF and bFGF, which will encourage cell migration towards the wound site. Over the past years, growth factor and cell-based therapies were developed to improve WH [43, 45]. Unfortunately, clinical trials of single growth factor delivery treatments resulted in poor outcomes [6, 46] suggesting that replacement of a single factor is not sufficient to ameliorate WH. Instead, a combined treatment composed of a pool of growth factors may provide a more integrated method for a therapeutic approach to actively improve WH [43]. Accordingly, we show that AMG extract carries bioactive molecules in the amount that is sufficient to promote cell motility such as IGF-I (4 ng/mL) and bFGF (0.5 ng/mL), which have shown to reach ED50 in human cells [47]. This new growth factor-dependent AMG mechanism may act in parallel and reinforce the previously described MSCs-based mechanisms within the AMG solution [1, 4]. Collectively we show that AMG treatment enhances the efficiency of the healing process through the delivery of growth factors, which favor the tissue regeneration and repair.

Transcriptome analysis of AMG-treated fibroblasts showed upregulation of the MAPK/ERK signaling pathway. Modulation of MAPK/ERK signaling pathway has been reported to induce changes in cell proliferation [31], cell migration [48] as well as in regulating tissue repair functions in fibroblasts [49]. However, downstream functions of this pathway during WH are still controversial. In line with the literature, our study demonstrates that AMG leads to an ERK-dependent increased cell migration. Consistent with enhanced fibroblast motility, we showed that granulation tissue formation and angiogenesis are induced by AMG in vivo, and they are all diminished upon topical application of MEK inhibitor trametinib, highlighting the essential role of the ERK pathway in AMG-mediated WH in vivo.

Our study shows that AMG treatment increases gene expression and enzymatic activity of MMPs. These enzymes play pivotal proteolytic roles in several biological processes, including WH [50]. MMP-1a, MMP3, and MMP9 are known as major chemokine regulators during this process [51], while fibroblast expression of *Mmp13* regulates myofibroblast functions and granulation tissue formation [52]. Interestingly, *Mmp3*- and *Mmp13*-deficient mice showed delayed WH and wound contraction failure, demonstrating their essential role in this process [53, 54]. Our work revealed a specific MMPs signature associated with WH in AMG-treated cells, as *Mmp1a*-*1b*-9-*10*-*12* and *13* are highly upregulated and enzymatically active upon the treatment. Consistent with these findings, an upregulation of *MMP1*, *MMP9*, and *MMP10* expression levels was observed in MG-treated keratinocytes. Moreover, we coupled MMPs activation with the acceleration of scratch closure (Fig. 4d, e), in line with other studies [10]. Mechanistically, we reveal that MMPs inhibition—through actinonin—reduces in vitro scratch closure, demonstrating their important role in the AMG molecular mechanism. Similarly, we demonstrate that the downregulation of MMPs is MEK inhibitor dependent in AMG-treated cells. Differently from the in vivo evidence, *Mmp12* expression remained unchanged upon MEK inhibition, which may reflect experimental differences in time of treatments (12 h in vitro treatment versus 8 days treatment in vivo). Furthermore, we cannot exclude the involvement of alternative or parallel signaling pathways, which may regulate distinct AMG-dependent cellular functions in in vivo WH.

The results of our study highlight the power of AMG treatment to enhance WH by promoting migration of fibroblasts and keratinocytes. We reveal that the molecular key of this new treatment involves a cascade of growth factors, which may initiate the activation of MAPKs and ERK leading to the induction of MMPs transcription and enzymatic activation.

Taken together, we demonstrate that AMG extract retains a regenerative capacity by improving the whole WH process and we provide insights into the AMG molecular mechanism opening new perspectives in the treatment of tissues injuries.

### Data and materials availability

RNA sequencing data are deposited in GEO Datasets under accession number GSE123829.

## Supplementary information

Figure S1

Figure S2

Figure S3

Figure S4

Figure S5

Figure S6

Figure S7

Figure S8

Figure S9

Table S1

Table S2

Table S3

Table S4

Table S5

Supplementary information
