# Supplementary figures and images for: Autologous micrograft accelerates endogenous wound healing response through ERK-induced cell migration
Source: Cell Death Differ. 2019 Oct 25;27(5):1520–38. doi: 10.1038/s41418-019-0433-3 (PMC7206041; doi:10.1038/s41418-019-0433-3)

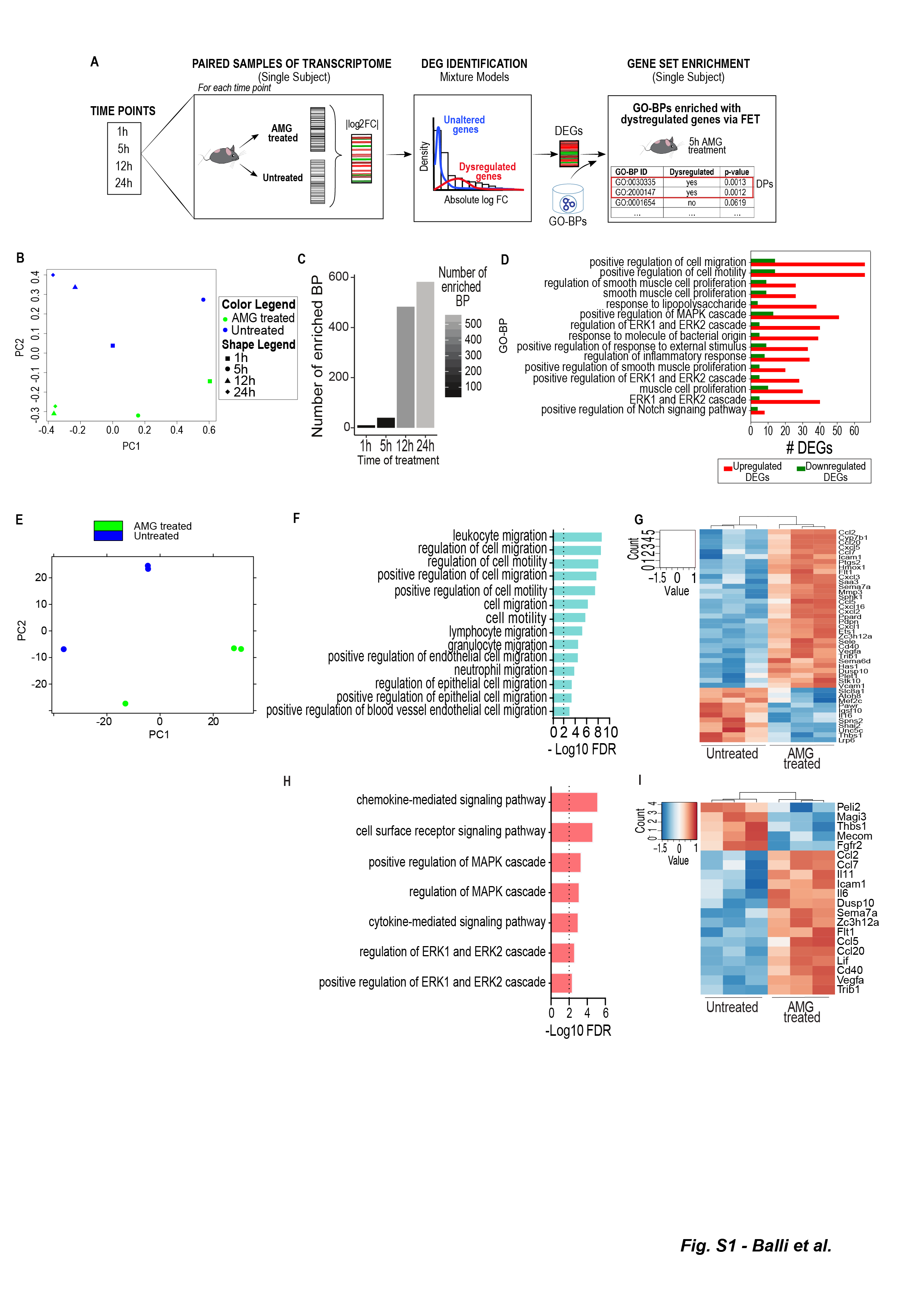

Supplement: Supplementary file 1 — Figure S1 [file 41418_2019_433_MOESM1_ESM.png]

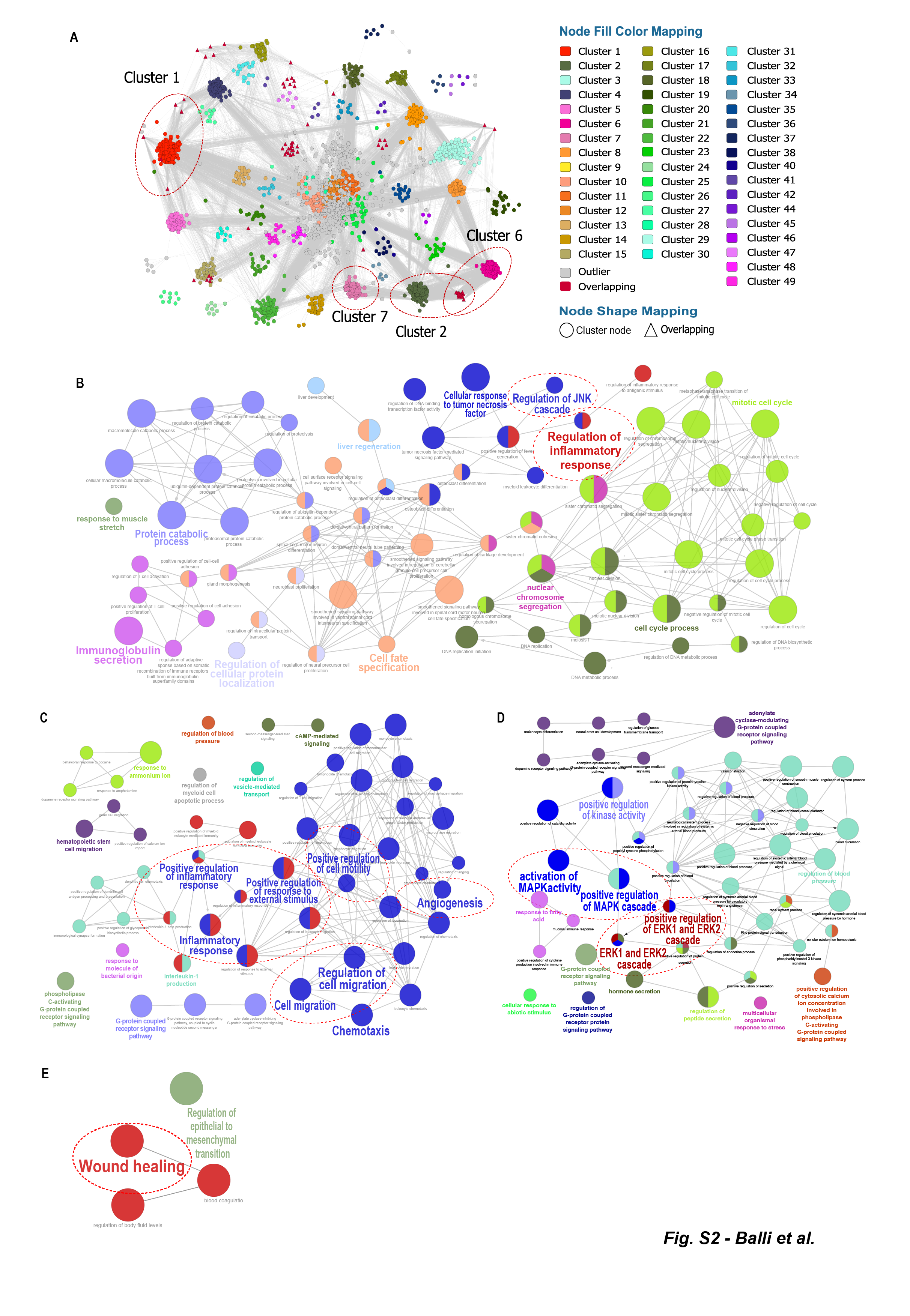

Supplement: Supplementary file 2 — Figure S2 [file 41418_2019_433_MOESM2_ESM.png]

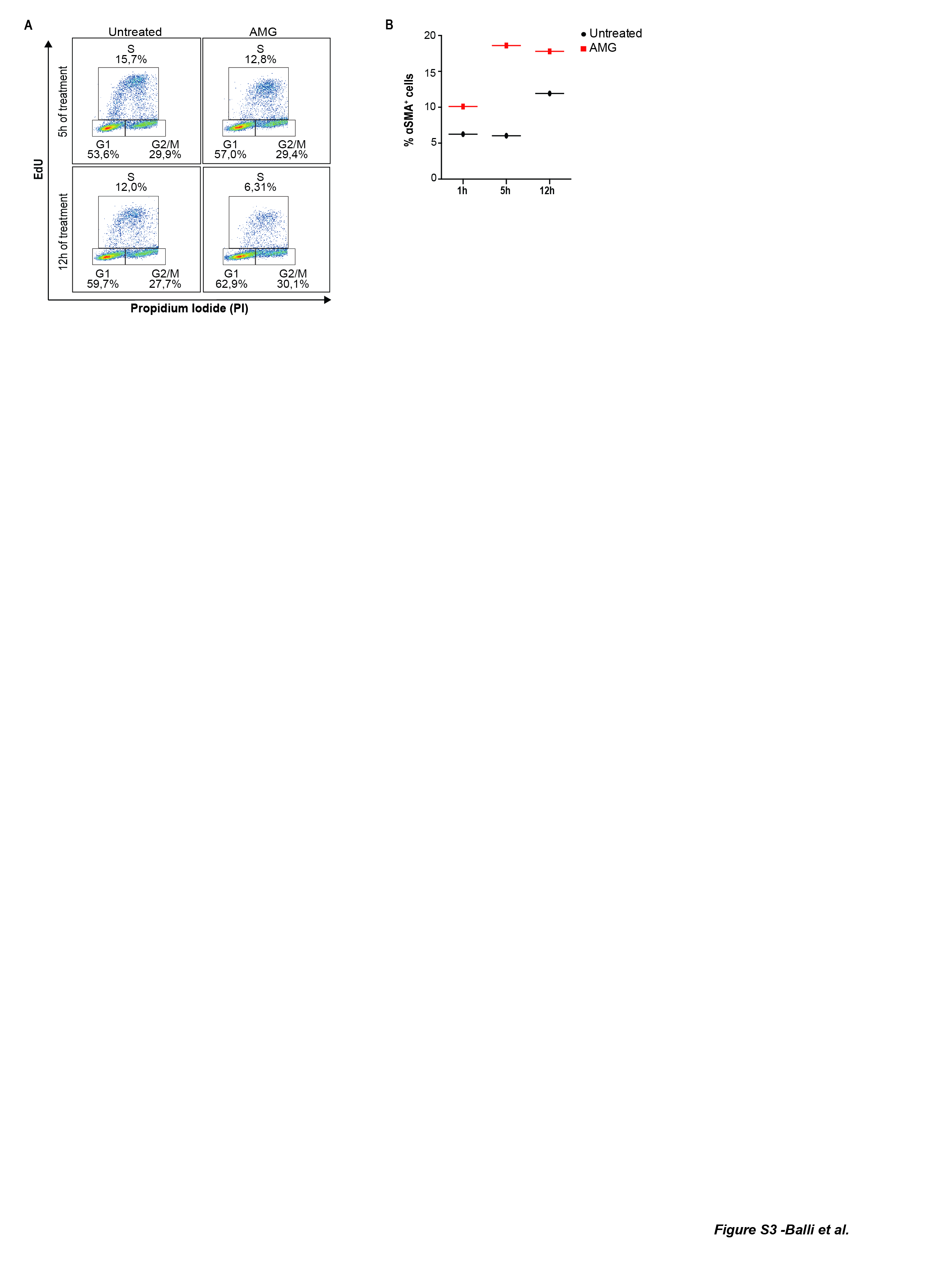

Supplement: Supplementary file 3 — Figure S3 [file 41418_2019_433_MOESM3_ESM.png]

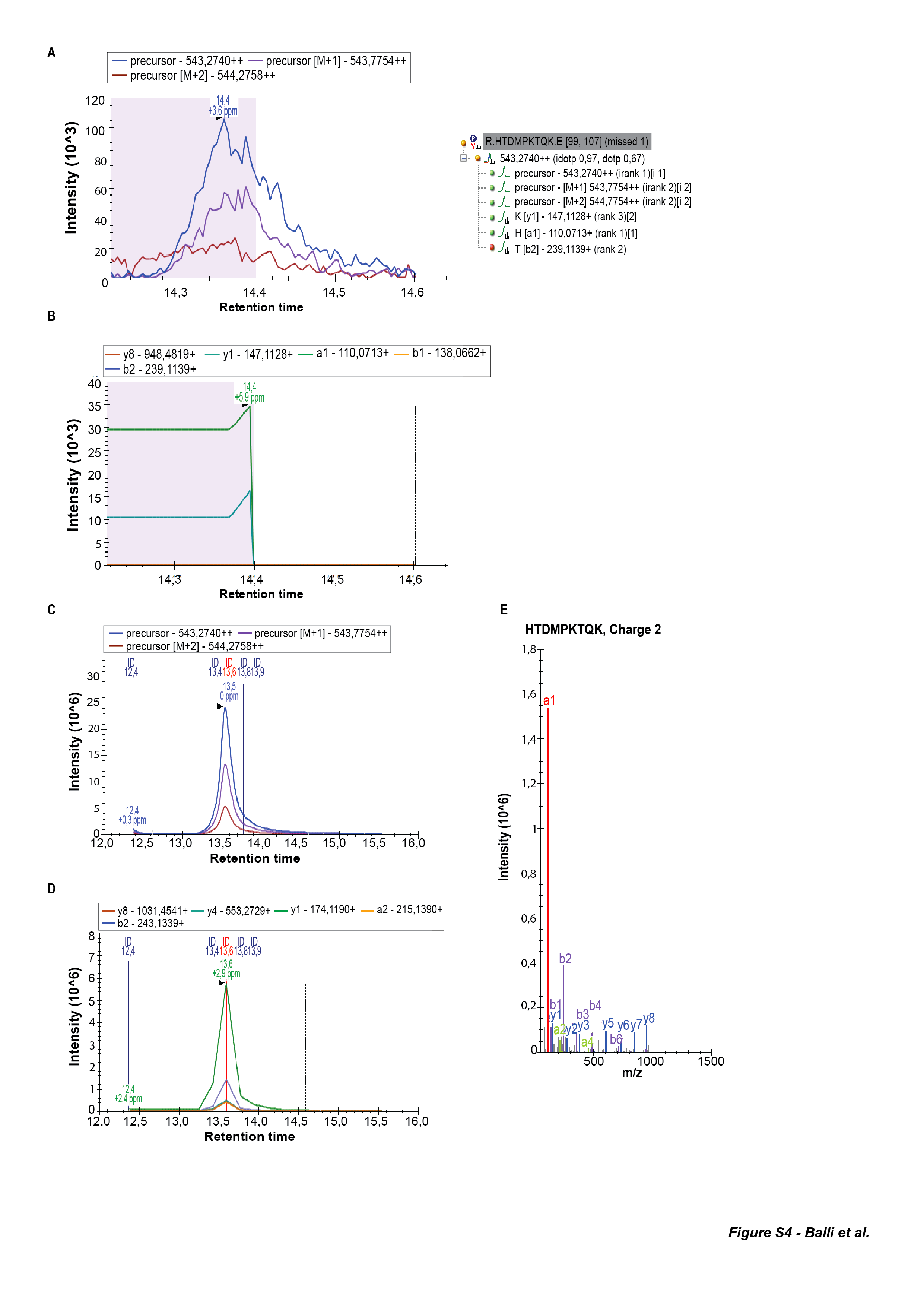

Supplement: Supplementary file 4 — Figure S4 [file 41418_2019_433_MOESM4_ESM.png]

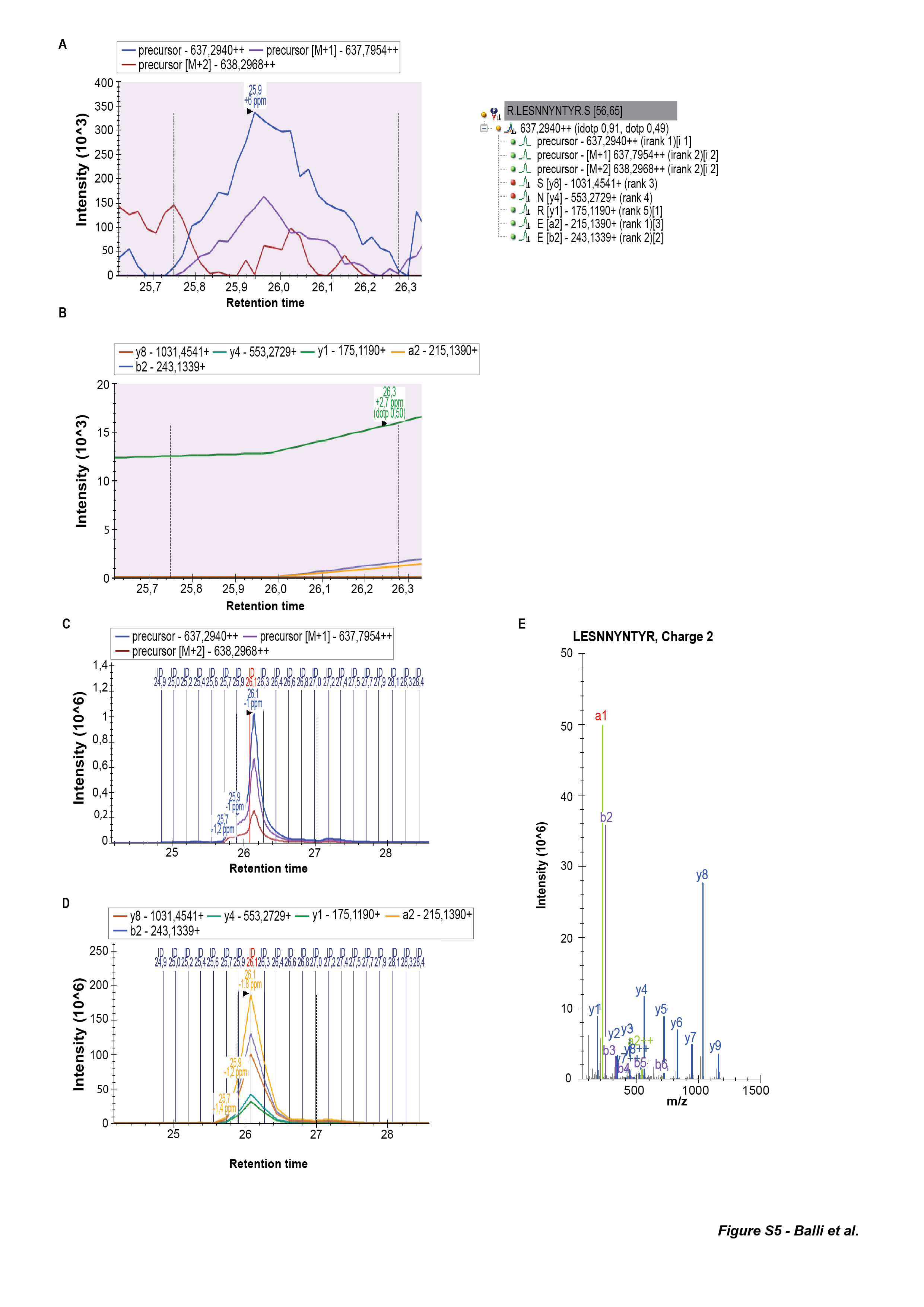

Supplement: Supplementary file 5 — Figure S5 [file 41418_2019_433_MOESM5_ESM.png]

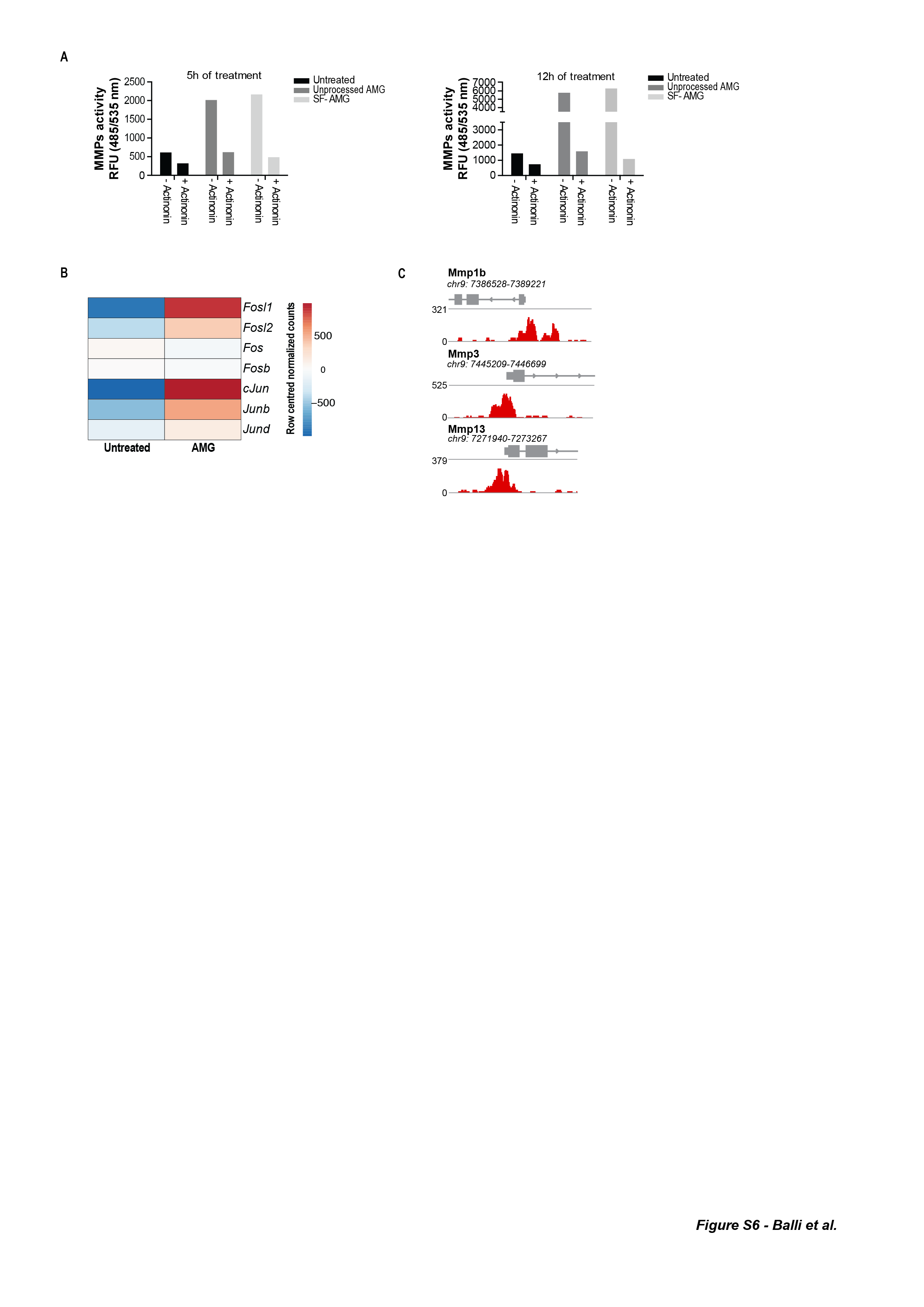

Supplement: Supplementary file 6 — Figure S6 [file 41418_2019_433_MOESM6_ESM.png]

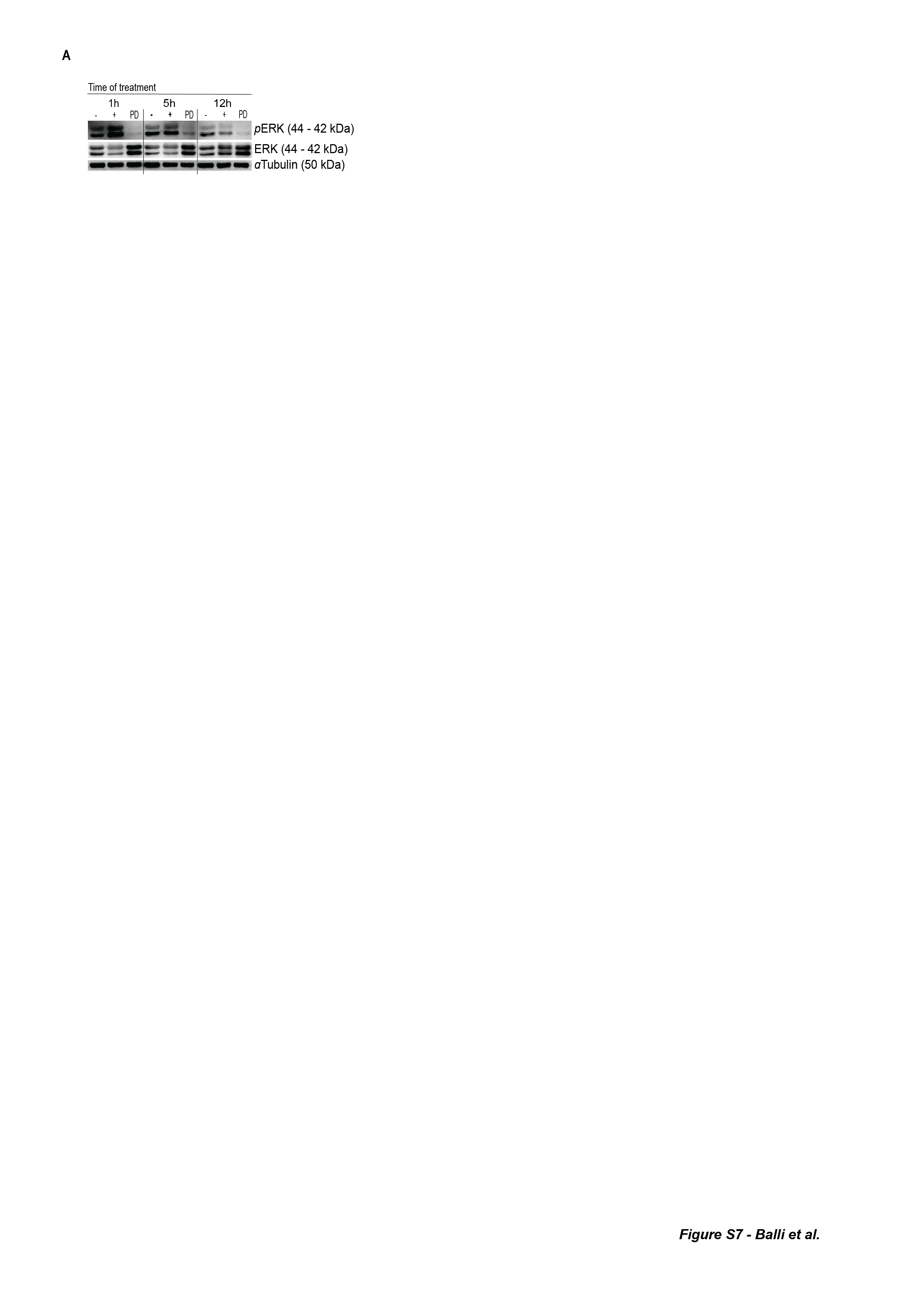

Supplement: Supplementary file 7 — Figure S7 [file 41418_2019_433_MOESM7_ESM.png]

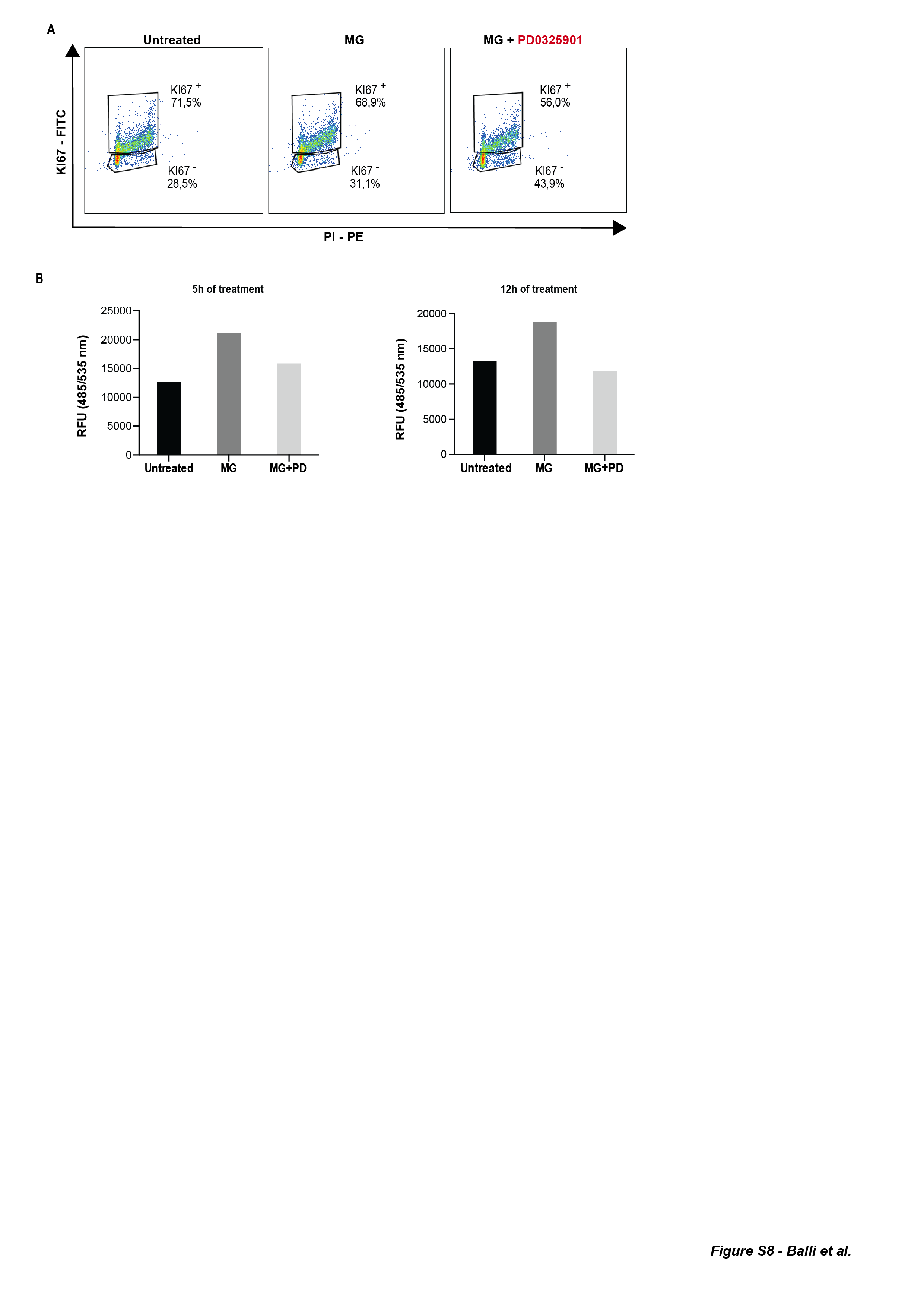

Supplement: Supplementary file 8 — Figure S8 [file 41418_2019_433_MOESM8_ESM.png]

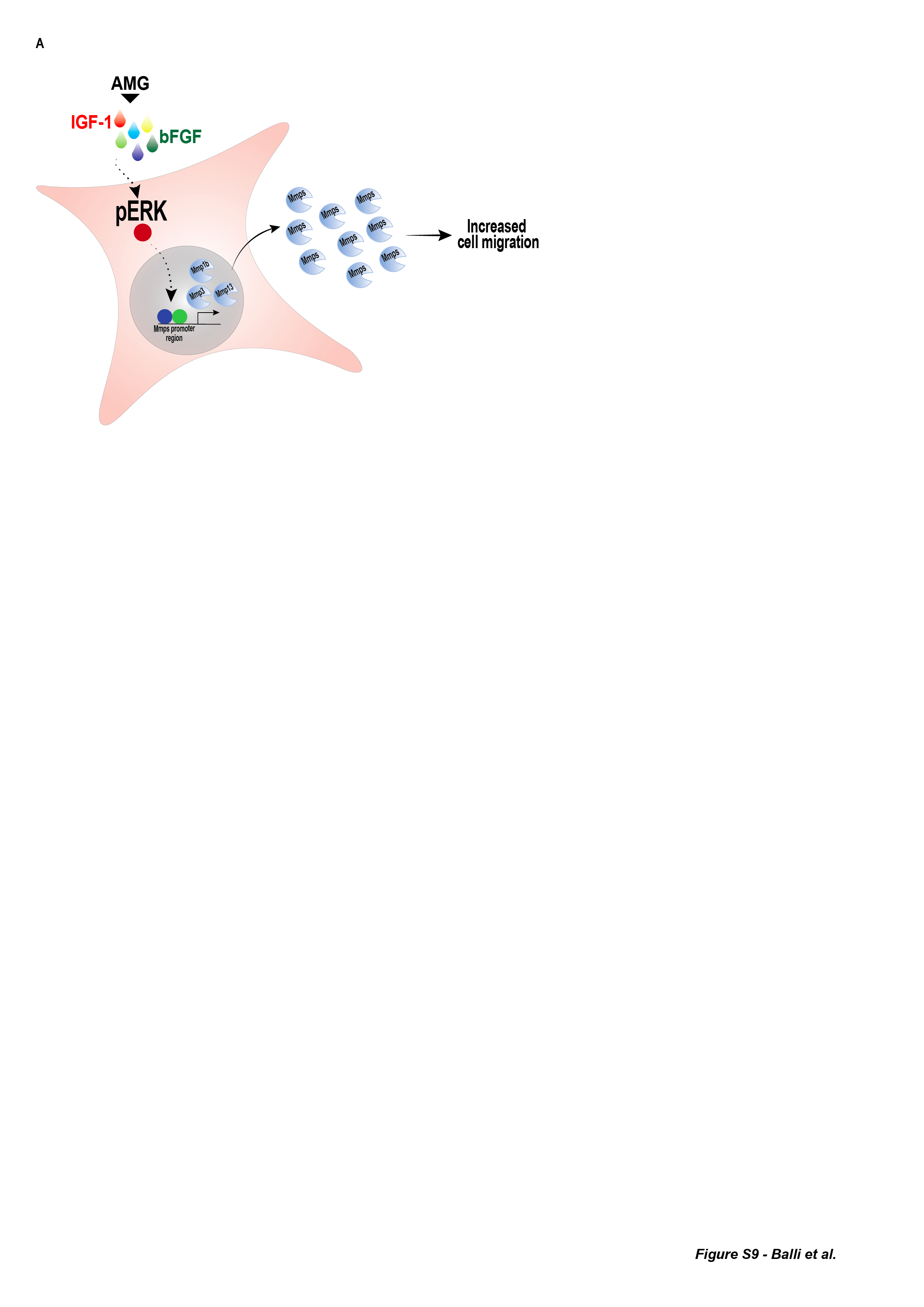

Supplement: Supplementary file 9 — Figure S9 [file 41418_2019_433_MOESM9_ESM.png]
