# Supplementary material for: Autologous micrograft accelerates endogenous wound healing response through ERK-induced cell migration
Source: Cell Death Differ. 2019 Oct 25;27(5):1520–38. doi: 10.1038/s41418-019-0433-3 (PMC7206041; doi:10.1038/s41418-019-0433-3)
Supplement: Supplementary file 15 — Supplementary information [file 41418_2019_433_MOESM15_ESM.docx]

**Supplementary information**

**Library Peptide Information**

| Protein | Sequence | Peptide Length | m/z | z | start | end |
| --- | --- | --- | --- | --- | --- | --- |
| Insulin-like growth factor 1 | HTDMPKTQK | 9 | 543,274 | 2 | 13,2 | 14,6 |
| Fibroblast growth factor | LESNNYNTYR | 10 | 637,294 | 2 | 25,7 | 27,2 |

Supplementary Figure Legends

Fig. S1. Cell migration and MAPK cascade activation as main contributors to the AMG effect. (A) Schematic overview of the single subject analysis performed via N-of-1 MixEnrich pathways on untreated vs AMG-treated murine primary fibroblasts. (B) In the figure are shown the fist two principal components (PC 1 and PC2) resulting from the Principal component analysis (PCA) performed on the pre-processed RNA-seq data. Color and shape indicate sample type (AMG-treated or untreated) and the different time points (1h, 5h, 12h, 24h), respectively. The two classes are well separated with the first two principal components as new features. (C) Number of enriched GO terms associated with biological processes for each time point of AMG treatment. (D) Enriched dysregulated pathways upon 5h of AMG treatment obtained from Gene Ontology terms related to Biological Processes (GO-BP) (FDR<0.05). (E) Principal component analysis (PCA) showing transcriptome differences caused by the AMG treatment (5 hours) (N=3). (F) DEGs-associated enriched GO-BP selected at FDR < 0.05 and presented using GraphPad Prism 6. (G) Heat map showing up-regulated genes involved in process related with cell migration and cell motility (GO:0030334, GO:0030335, GO:0016477, GO:0010632, GO:0010634, GO:2000145, GO:2000147 and GO:0048870). (H) DEGs-associated GO terms linked with positive regulation of MAPK activity and ERK1/2 cascade were assessed as targets of the AMG treatment. (I) Heatmap showing up-regulated genes involved in process related with ERK1/ERK2 and MAPK (GO:0070372, GO:0070374, GO:0043410, GO:0043408).

Fig. S2. AMG treatment actively triggers important WH-associated signaling pathways. (A) Clusters have been obtained through ClusterONE and presented with different colors. Among the obtained 49 significant, clusters including important WH-related biological processes have been selected and presented separately. (B) Cluster 1: Positive regulation of the inflammatory response, including regulation of the JNK cascade, tumor necrosis factor-mediated signaling pathways as well as regulation of DNA-binding transcription factor activity have been found as candidate targets of the AMG treatment. (C) Cluster 2: Positive regulation of cell motility, cell migration, chemotaxis, angiogenesis and regulation of response to external stimulus have been identified as candidate target of the AMG treatment. (D) Cluster 6: Positive regulation of kinase activity, including positive regulation and activation of both the MAPK activity and ERK1/2 cascade has been assessed as target of the AMG treatment. (E) Cluster 7: Regulation of the WH process is also referred as candidate of the AMG treatment.

Fig. S3. AMG cellular effects on *in vitro* cellular functions. (A) Representative FACS plots performed on 60-70% confluent cells upon 5 and 12h AMG treatments. Untreated cells were used as a control. Percentage of cell cycle phases was obtained upon EdU (5-ethynyl-2’-deoxyuridine) and PI (Propidium Iodide) stainings. (B) Percentage of normalized ɑ-SMA protein levels in AMG-treated cells. (N=1).

**Fig. S4. Quantification of AMG-derived Insuline-like growth factor I.** **(A)** Intensity of the peak areas of the parent ions of HTDMPKTQK. **(B)**. Intensity of the peak areas of the MSMS ions confirming the presence of the first and the last AA. All 3 precursor parents were detected and 2 of the 3 most intense MSMS ions were confirmed (green). **(C)** Intensity of the peak areas of the parent ions of HTDMPKTQK of the library peptide (120 ng). **(D)** Intensity of the peak areas of the MSMS ions indicating the 5 most intense peaks. **(E)** Complete MSMS spectrum of the HTDMPKTQK library peptide.

**Fig. S5: Quantification of AMG-derived basic Fibroblasts growth factor. (A)** Intensity of the peak areas of the parent ions of LESNNYNTYR. **(B)** Intensity of the peak areas of the MSMS ions confirming the presence of the second and the last AA. All 3 precursor parents were detected and 3 of the 5 most intense MSMS ions were confirmed (green). **(C)** Intensity of the peak areas of the parent ions of LESNNYNTYR of the library peptide (120 ng). **(D).** Intensity of the peak areas of the MSMS ions indicating the 5 most intense peaks. **(E).** Complete MSMS spectrum of the LESNNYNTYR library peptide

Fig. S6: Matrix metalloproteinase enzymatic activity inhibition in an *in vitro* model of WH. (A) MMP extracellular activity in the biological samples exposed to the AMG treatment. Enzymatic activity of all MMP members present in cell supernatant was fluorometrically detected. Signals were evaluated 30 minutes after starting the reaction using a microplate reader with a filter set of Ex/Em = 485/535. The fluorescence signal obtained from each sample was normalized on the substrate control. Groups were incubated or not with the MMP inhibitor – Actinonin – 20μM. Groups of wounded cells that did not receive any treatment were used as a control. Data are presented as RFU (Relative Fluorescence Units) as result of technical replicates. (B) *In vivo* expression of AP-1 family members upon AMG treatment. Data are presented as the average of three biological replicates. (C) Levels of FRA1 binding around MMPs promoter regions.

Fig. S7. ERK phosphorylation upon AMG-based treatment in an *in vitro* model of WH. (A) Representative western blotting showing protein level of phosphorylated and total ERK1/ERK2 obtained from wounded murine primary fibroblasts exposed to AMG treatment and in which we induced the inhibition of the MAPK signaling pathway (using the MEK inhibitor PD0325901 - 1 μM) for different time periods (1, 5 and 12h).

Fig. S8. MG-dependent effects on epithelial cells. (A) Representative FACS plots performed on 5h MG-treated keratinocytes in the presence or absence of MEK inhibitor PD0325901 (1 μM). Untreated cells were used as a control. Percentage of cell cycle phases was obtained upon KI67 and PI (Propidium Iodide) staining via flow cytometry (BD-Biosciences) and analyzed by FlowJo software. (B) Enzymatic activity of MMP members present in keratinocytes conditioned supernatant was fluorometrically detected. Data are presented as Relative Fluorescence Units (RFU). Signals were evaluated 30 minutes after starting the reaction using a microplate reader with a filter set of Ex/Em = 485/535. The fluorescence signal obtained from each sample was normalized on the substrate control. Groups of wounded cells that did not receive any treatment were used as a control.

Fig. S9. Overview of the AMG molecular mechanism. Application of AMG treatment both in *in vitro* and *in vivo* WH assays triggers to activation of both ERK signaling pathway and MMP expression and enzymatic activity. The WH-related pool of growth factors identified within the AMG may play a cardinal role in the activation of the MAPK cascade. Activation of the transcription factor may induce MMP transcription and translation. Altogether, these events lead to an increase in cell migration rate, accelerating the whole WH process.

Tables

Table S1. N-of-1 MixEnrich Pathways analysis upon AMG treatment.

Table S2. DEGs associated to AMG treatment exposure and gene ontology enrichment analysis.

Table S3. Identification of AMG targets: DEGs-related Hub nodes and cluster analysis of the AMG-mediated WH network.

Table S4. AMG-derived protein identification through Ultra Performance Liquid Chromatography - tandem Mass Spectrometry (UPLC-MS/MS).

Table S5. Primers for qRT-PCR.
